# Al_13_^−^ and B@Al_12_^−^ superatoms on a molecularly decorated substrate

**DOI:** 10.1038/s41467-022-29034-9

**Published:** 2022-03-14

**Authors:** Masahiro Shibuta, Tomoya Inoue, Toshiaki Kamoshida, Toyoaki Eguchi, Atsushi Nakajima

**Affiliations:** 1 Keio Institute of Pure and Applied Sciences (KiPAS), Keio University, 3-14-1 Hiyoshi, Kohoku-ku, Yokohama 223-8522 Japan; 2 Department of Chemistry, Faculty of Science and Technology, Keio University, 3-14-1 Hiyoshi, Kohoku-ku, Yokohama 223-8522 Japan; 3 Department of Physics, Graduate School of Science, Tohoku University, 6-3 Aramaki Aza-Aoba, Aoba-ku, Sendai 980-8578 Japan

**Keywords:** Physical chemistry, Electronic properties and materials

## Abstract

Aluminum nanoclusters (Al_*n*_ NCs), particularly Al_13_^−^ (*n* = 13), exhibit superatomic behavior with interplay between electron shell closure and geometrical packing in an anionic state. To fabricate superatom (SA) assemblies, substrates decorated with organic molecules can facilitate the optimization of cluster–surface interactions, because the molecularly local interactions for SAs govern the electronic properties via molecular complexation. In this study, Al_*n*_ NCs are soft-landed on organic substrates pre-deposited with *n*-type fullerene (C_60_) and *p*-type hexa-*tert*-butyl-hexa-*peri*-hexabenzocoronene (HB-HBC, C_66_H_66_), and the electronic states of Al_*n*_ are characterized by X-ray photoelectron spectroscopy and chemical oxidative measurements. On the C_60_ substrate, Al_*n*_ is fixed to be cationic but highly oxidative; however, on the HB-HBC substrate, they are stably fixed as anionic Al_*n*_^−^ without any oxidations. The results reveal that the careful selection of organic molecules controls the design of assembled materials containing both Al_13_^−^ and boron-doped B@Al_12_^−^ SAs through optimizing the cluster–surface interactions.

## Abstract (web summary)

Anionic aluminium clusters are promising candidates for the fabrication of superatom-assembled nanomaterials. Here, the authors report enhanced stability for Al_13_^−^ and boron-doped B@Al_12_^−^ on a molecularly decorated *p*-type organic substrate.

## Introduction

Through the deposition of size-selected atomic clusters consisting of a few to thousands of atoms on well-defined substrates, nanostructured surfaces can be produced through bottom-up fabrication, which is a promising method for creating low-dimensional nanomaterials with atomic-scale structural precision^1–4^. The properties of functionalized nanostructured surfaces can be controlled by designing cluster–surface interactions, which facilitates a nanoscale approach to developing nanomaterial-based modified electrodes for application in electrochemistry^5^. The cluster–surface interaction is a fundamental characteristic of such nanostructured materials^3,6^, and has been a focus in the preparation of heterogeneous catalysts through control of the physical and chemical properties, size, and dimensionality^7–10^. For example, Haruta indicated the importance of choosing a substrate in enhancing the catalytic activity of gold (Au) nanoparticles for low-temperature CO oxidation^7^. In addition, the substrate acidity has been reported to control the catalytic activity of size-selective platinum (Pt) clusters^10^. In these studies, localized cluster–surface interactions are enhanced using metal oxide substrates^8,9^ to avoid the generation of weakly bound nanoclusters (NCs) on a clean surface, because these NCs generally behave as a two-dimensional gas, ultimately resulting in aggregation^6^.

Interactions that take place through charge transfer (CT), or more explicitly, electron transfer^11^, are important in chemical reactions between two reactant molecules since they lead to the formation of intermolecular CT complexes that exhibit a new electronic transition known as a CT band^12^. Their segregated stacking can lead to molecular electrical conductivity, including superconductivity^13,14^. Such CT processes play an important role in cluster–surface interactions. More specifically, due to the CT interactions with pre-deposited organic molecules on a substrate, the NCs can exist in a monodisperse state on the surface^15,16^.

Among various gas phase NCs and their characteristic functionalities explored during the past several decades, NCs formed with a highly symmetrical geometry and an electronically closed shell are known as “*superatoms*” (SAs), which mimic the chemical properties of atoms with clusters^17–25^. In particular, anionic aluminum (Al) NCs with 13 atoms, i.e., Al_13_^−^, are promising candidates for the fabrication of SA assembled nanomaterials^26–31^, because Al_13_^−^ simultaneously satisfies both icosahedral packing and the electronic shell closing^32,33^ of 40 electrons as (1S)^2^(1P)^6^(1D)^10^(2S)^2^(1F)^14^(2P)^6^, thereby facilitating the bottom-up fabrication of nanostructures with desired functionalities, similar to the case of building nanoblocks^34–36^.

In this study, we show that the choice of organic substrate can allow molecular control of the CT interactions at the cluster–surface interface and stabilize SAs on the surface. Since the localized interactions between the pre-decorated organic molecules and the deposited NCs are enhanced compared to those of a clean bulk metal or semiconductor substrate, the organic substrate is key to immobilization of the deposited NCs, in which the NC aggregation caused by two-dimensional gas behaviors is suppressed^16,25^. Thus, we deposit Al_13_^−^ and boron-doped B@Al_12_^−^ SAs^17,26,37,38^ on organic substrates of *n*-type C_60_ and *p*-type hexa-*tert*-butyl-hexa-*peri*-hexabenzocoronene (HB-HBC, C_66_H_66_ (see Supplementary Fig. 1 and Supplementary Note 1)). Spectroscopic characterization by X-ray photoelectron spectroscopy (XPS) and oxidative reaction measurements of the Al_13_^−^ and B@Al_12_^−^ SAs on the organic substrates are then conducted to reveal that superatomic behavior can be observed on the *p*-type organic substrates through CT interactions.

## Results

### Charge state of the Al_*n*_ NCs on *n*-type C_60_ and *p*-type HB-HBC substrates

Through magnetron sputtering (MSP) of the Al targets, the generated Al_*n*_^−^ NCs possessed a mass-to-charge ratio (*m/z*) predominantly in the range of 200−800 (see Supplementary Fig. 2). With an ion current of 300 pA, samples containing 2.9 × 10^13^ mass-selected NCs (~0.6 monolayers (MLs)) could be prepared within 3 h (see “Methods” section and Supplementary Note 2). The morphology of the deposited NCs on the organic substrate was confirmed by scanning tunneling microscopy (STM) imaging^16,25^, wherein the SAs were found to be monodispersively immobilized without aggregation (see Supplementary Fig. 3).

**Fig. 1:**
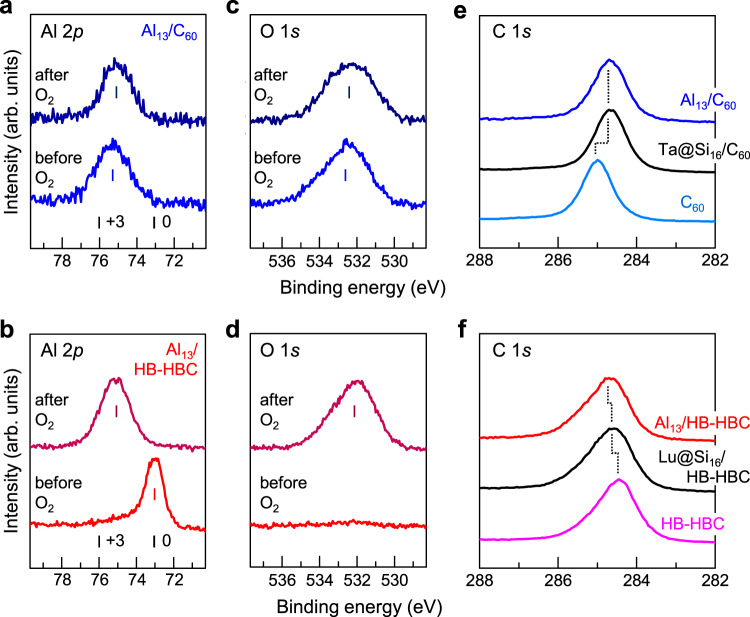
XPS spectra for Al_13_ on the C_60_ and HB-HBC substrates. **a**–**d** XPS spectra around the Al 2*p* core levels for **a** Al_13_ on C_60_ and **b** Al_13_ on HB-HBC before (lower) and after (upper) O_2_ exposure (at 5 × 10^10^ Langmuir (L = 1.33 × 10^−4^ Pa·s)), along with **c, d** the O 1*s* spectra for each state. **e**, **f** The XPS spectra around the C 1*s* core level for the underlying **e** C_60_ and **f** HB-HBC are also shown for the deposition of Al_13_ and Ta@Si_16_ or Lu@Si_16_. Reference binding energies (BEs) of Al 2*p*_3/2_ for the bulk Al (Al^0^ and Al^3+^) and O 1*s* (O^2−^) are marked by vertical bars. The BEs for Al 2*p* show zerovalent Al^0^ only for Al_13_ on the HB-HBC substrate before O_2_ exposure, while the other BEs are in the vicinity of Al^3+^. After the deposition of 0.6 ML Al_13_ (blue) or Ta@Si_16_ (black) on C_60_, the C 1*s* peak in (**e**) shifts by ~0.3 eV toward a lower BE from that before deposition (light blue), indicating the presence of an anionic C_60_^−^ state. After the deposition of 0.6 ML Al_13_ (red) or Lu@Si_16_ (black) on HB-HBC, the C 1*s* peak in (**f**) shifts by ~0.25 eV toward a higher BE from that before deposition (pink), indicating the presence of a cationic HB-HBC^+^ state.

Figure 1a, b show the XPS spectra around Al 2*p* core levels for (a) Al_13_ on C_60_ and (b) Al_13_ on HB-HBC before (lower) and after (upper) O_2_ exposure, respectively. The binding energies (BEs) of Al 2*p*_3/2_ for the bulk Al (Al^0^) and oxidized Al (Al^3+^) have been previously reported (marked by vertical bars in the figure)^39^. As can be seen, without O_2_ exposure, the Al atoms on the C_60_ substrate are completely oxidized, while the Al atoms on HB-HBC are not oxidized. Following O_2_ exposure, the Al atoms on C_60_ remain unchanged, while Al atoms on HB-HBC are oxidized to Al^3+^. As shown in Fig. 1c, d, the corresponding O 1 *s* component can be observed in the lower trace of Fig. 1c even without O_2_ exposure. These results show that the Al_13_ NCs present on the C_60_ substrate are so reactive that the nascent NCs are oxidized after deposition by some residual gas in the vacuum chamber (<10^−5^ Pa) during the deposition process.

The contrasting oxidation behavior of these two systems correlates well with the C 1*s* XPS peaks from the underlying C_60_ or HB-HBC on highly oriented pyrolytic graphite (HOPG), where the C 1*s* signals are mainly derived from the topmost molecular layer (Fig. 1e, f). As shown in Fig. 1e, after the deposition of Al_13_ on C_60_, the C 1*s* peak shifts toward a lower BE by ~0.30 eV. Although Al_13_ is nascently oxidized, the shift to a lower BE shows that an anionic C_60_^−^ state is formed by Al_13_ oxides through a CT interaction^16,40^; the degree of shift corresponds well to the formation of C_60_^−^ as reported in the literature^41^ (see Supplementary Note 3 and Supplementary Fig. 4). A similar C 1*s* shift has been reported when the alkali-like Ta@Si_16_ SA^25^ is deposited on C_60_, wherein a shift attributable to Ta@Si_16_^+^C_60_^−^ is observed^40,41^, as denoted in Fig. 1e. More quantitatively, when the C_60_-derived C 1*s* peak is deconvoluted into two peak components corresponding to C_60_ alone (non-interacted) and bound with the Al_*n*_ oxide (interacted), the BE of the interacted C_60_ peak is 0.33 eV lower than that of non-interacted C_60_ (Supplementary Fig. 4). In addition to Al_13_, the Al_7_ NCs deposited on C_60_ is nascently oxidized, as can be observed from the Al 2*p* XPS spectrum, although Al_7_^+^ is regarded to complete the 2S shell (i.e., 20 e^−^)^21^. In contrast, after the deposition of 0.6 MLs of Al_13_ on the HB-HBC substrate, the C 1*s* peak shown in Fig. 1f shifts toward a higher BE by ~0.25 eV. Since a similar behavior can be observed for the deposition of the halogen-like Lu@Si_16_ SA^25^ onto HB-HBC, this shift suggests the formation of a cationic HB-HBC^+^ state, and in turn, an Al_13_^−^/HB-HBC^+^ CT complex.

In addition to Al_13_^−^, all Al_*n*_^−^ NCs (*n* = 7–24) can be size-selectively deposited onto C_60_ and HB-HBC substrates. More specifically, the Al 2*p* XPS spectra show that these Al_*n*_ NCs were successfully deposited onto HB-HBC without undergoing any oxidation reactions (see Supplementary Fig. 5). However, complete oxidation was observed for the Al_*n*_ NCs deposited on C_60_. It should be emphasized that this contrast in the reactivity of Al_*n*_ results from the different types of organic substrate molecules, i.e., *n*-type and *p*-type for C_60_ and HB-HBC, respectively. In the Al 2*p* XPS spectra for the Al_*n*_ NCs on HB-HBC, peaks were observed in the range of 73.0–73.2 eV, which is close to the peak position for bulk Al (i.e., 73.0 eV)^39^ (see Supplementary Fig. 6). In addition, the small size dependence is consistent with that in the Al 2*p* core-level BEs for Al_*n*_^+^ (*n* = 12–15) obtained from the soft X-ray photoionization efficiency curves^42^. More precisely, the charge states of the Al atoms for the deposited Al_*n*_ NCs can be discussed in terms of their Al 2*p* peak positions; the BEs of Al 2*p* for all Al_*n*_ NCs are slightly higher than that of the bulk Al (zerovalent Al^0^), suggesting that the Al_*n*_ NCs on HB-HBC are anionic rather than neutral. Recently, Kambe et al. have reported the Al 2*p* XPS spectra for several Al_*n*_ species (*n* = 4, 12, 13, 28, and 60) synthesized with dendrimers^43^, and they revealed a size-dependent behavior in the Al 2*p* XPS spectra from 71.2 (*n* = 4) to 72.3 eV (*n* = 13) along with a particular shift of more than 0.6 eV between *n* = 12 and 13. However, our Al 2*p* spectra exhibit a cluster-size dependence within only 0.3 eV for *n* = 7–24, and no particular peak shift can be observed around *n* = 13. It should be noted here that the peaks in the C 1*s* XPS spectra for the Al_*n*_ NCs on the C_60_ and HB-HBC/HOPG substrates exhibit a similar contrast shift; namely a decrease in the BE for the Al_*n*_ NCs on C_60_ (−0.30 eV) and an increase in the BE for the Al_*n*_ NCs on HB-HBC (+0.25 eV), with a small size-dependent shift being observed (see Supplementary Fig. 7).

### Oxidative reactivity of Al_*n*_ on the HB-HBC substrate

**Fig. 2:**
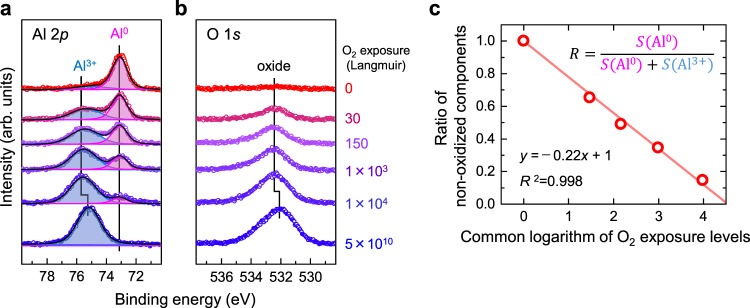
Oxidative behaviors in the Al 2*p* and O 1*s* XPS spectra for Al_13_ on the HB-HBC substrate. **a**, **b** XPS spectra around **a** Al 2*p* and (**b**) O 1*s*. With increasing O_2_ exposure (from top red to bottom blue), the intensity of the zerovalent component (Al^0^) decreases, while those of the oxidized component (Al^3+^) and the O 1*s* component increase accordingly. **c** The oxidative reactivity is evaluated by the slope of the dependence against the logarithmic O_2_ exposure amount from 0 L to 1 × 10^4^ L. At the highest exposure of 5 × 10^10^ L, the Al 2*p* peak shifts to a lower BE, likely due to a structural change relevant to the phase transition of aluminum oxide (see the main text for further details).

As shown in Fig. 1b, the Al_*n*_ NCs deposited on HB-HBC are oxidized upon O_2_ exposure, and the oxidative rates are dependent on the NC size. Figure 2 shows the Al 2*p* XPS spectra for the Al_13_ on HB-HBC at several different O_2_ exposure amounts (i.e., 0–5 × 10^10^ L), where the O_2_ exposure amounts (in Langmuir units, L = 1.33 × 10^−4^ Pa·s)) are noted on the right-hand side in of the figure. With increasing the amount of O_2_ exposure, the intensity of the peak corresponding to the zerovalent Al^0^ component decreases, while that of the oxidized component Al^3+^ increases along with that of the O 1*s* component. The oxidative reactivity can therefore be quantitatively evaluated on the basis of its dependence on the O_2_ exposure amount from 0 to 1 × 10^4^ L. It should be noted here that at 1 × 10^4^ L O_2_, the Al^0^ component survives only in the case of *n* = 13 (Supplementary Fig. 5), which is peculiarly unreactive compared to NCs of other sizes and with Al single crystal surfaces, which are completely oxidized when exposed to 400 L O_2_ at room temperature^44^. Furthermore, both the Al 2*p* and O 1*s* peaks shift to a lower BE when the O_2_ exposure amount is increased from 1 × 10^4^ to 5 × 10^10^ L, thereby implying that a structural change relevant to a phase transition from amorphous to crystalline Al_2_O_3_ takes place, such as the formation of α- or γ-Al_2_O_3_^45^.

The chemical reactivity of the Al_*n*_ NCs toward O_2_ gas was then evaluated based on the oxidation rate, *O*_Al*n*_, which is a simple index for investigating the size-dependent behavior of the oxidation reaction. More specifically, the peak area ratio, *R*_Al*n*_, of the non-oxidized component (${{{{{{\rm{S}}}}}}}_{A{l}^{0}}$) to the oxidized component (${{{{{{\rm{S}}}}}}}_{A{l}^{3+}}$) for the Al 2*p* spectra is plotted against the logarithm of the O_2_ exposure amount in L (log_10_ O_2_), and the linear slope is evaluated as *O*_Al*n*_, where *R*_Al*n*_ is expressed as follows:

1 $${R}_{A{l}_{n}}=\,\frac{{S}_{A{l}^{0}}}{{S}_{A{l}^{0}}+\,{S}_{A{l}^{3+}}}$$

In this analysis, the oxidation of Al atoms by O_2_ is modeled in terms of the dissociative adsorption of O_2_ on a single crystal Al surface, whose XPS peak appears at a BE close to that of the Al^3+^ component (i.e., 75−76 eV)^44^:

2 $${{{{{{\rm{A}}}}}}{{{{{\rm{l}}}}}}}_{n}\cdot {x{{{{{\rm{O}}}}}}}_{2}+{{{{{{\rm{O}}}}}}}_{2}\to {{{{{{\rm{Al}}}}}}}_{n}\cdot ({x+1}){{{{{\rm{O}}}}}}_{2},\,\left(x=0,\,1,\,2,\cdots \right)$$

The oxidation rates, *O*_Al*n*_, are evaluated by considering the conversion of 1 L → 1*s* because the exposure amount can be converted to the corresponding reaction time for elementary reactions. The intersection of the linear line with the x-axis in Fig. 2c gives the O_2_ exposure amount of *V*_Al13_(O_2_) that is required to completely oxidize the Al_*n*_ NCs; for Al_13_, 3.45 × 10^4^ L O_2_ is obtained as the value of *V*_Al13_(O_2_). The higher the reactivity, the smaller the quantity of oxygen required to completely oxidize the Al_*n*_ NCs; for example, *V*_Al*n*_(O_2_) at *n* = 12 is 2.36 × 10^3^ L O_2_, thereby showing that Al_12_ is 14.6 times more reactive than Al_13_ (further details regarding the *O*_Al*n*_ and *V*_Al*n*_(O_2_) values can be found in Supplementary Note 4 and Supplementary Table 1).

**Fig. 3:**
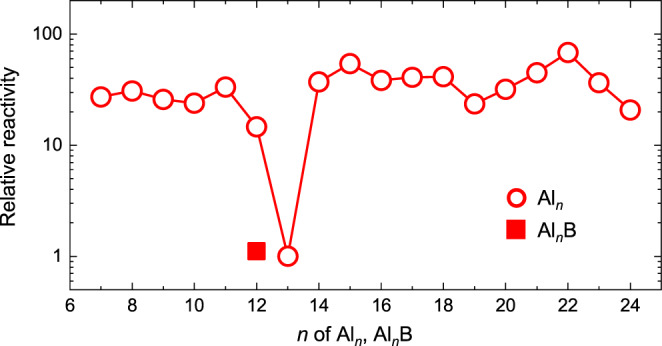
Size dependent relative reactivities of Al_*n*_ (*n* = 7–24) and B@Al_12_ on the HB-HBC substrate against the O_2_ exposure. The oxidative reactivity rates for Al_*n*_ on the HB-HBC substrate are plotted (red open circles), and a clear local minimum is observed at *n* = 13 along with a small minimum at *n* = 19, while there is no apparent local minimum at *n* = 23. B@Al_12_ shows a low oxidative reaction rate (red solid square), similar to that of Al_13_.

Figure 3 shows the size dependence of the oxidative rates on the Al_*n*_ NCs (*n* = 7–24) deposited on the HB-HBC substrate, where the relative reactivity is evaluated by dividing the *V*_Al*n*_(O_2_) value at *n* = 13 by each individual *V*_Al*n*_(O_2_) value. A local minimum is clearly found at *n* = 13, and a small local minimum is also found at *n* = 19, while an even–odd alternation relevant to spin conservation^46^ observed in the gas phase reaction^17,30,47,48^ is not obvious. According to previous experimental and theoretical works^17,30,38,49–51^, electronically stabilized Al_*n*_ anions should appear at *n* = 19 and 23 as well as at *n* = 13. Since its interactions with HB-HBC induces an anionic character in the deposited Al_*n*_ NCs, Al_13_ and Al_19_ complete their 2P (40 e^−^) and 1G (58 e^−^) shells, respectively. However, such stabilization was not observed for Al_23_ despite this species completing its 3S (70 e^−^) shell (see Fig. 3), and this was attributed to the fact that Al_23_ is geometrically deformed on the substrate owing to its relatively low rigidity having structural *C*_s_ symmetry^50–52^.

### Oxidative reactivity of B@Al_12_ on the HB-HBC substrate

The structural rigidity of icosahedral Al_13_^−^ is demonstrated by the boron (B) doped B@Al_12_^−^ SAs. Boron belongs to the same group as Al in the periodic table, and it has been reported B@Al_12_^−^ can be preferentially formed as an SA both experimentally and theoretically because the isoelectronic and geometrically small B atom facilitates relaxation of the icosahedral geometric strain when used as a central atom^37,38,53,54^. Thus, using a B-mixed Al target, B@Al_12_^−^ was formed by MSP and was deposited onto the C_60_ and HB-HBC substrates (Supplementary Fig. 8).

**Fig. 4:**
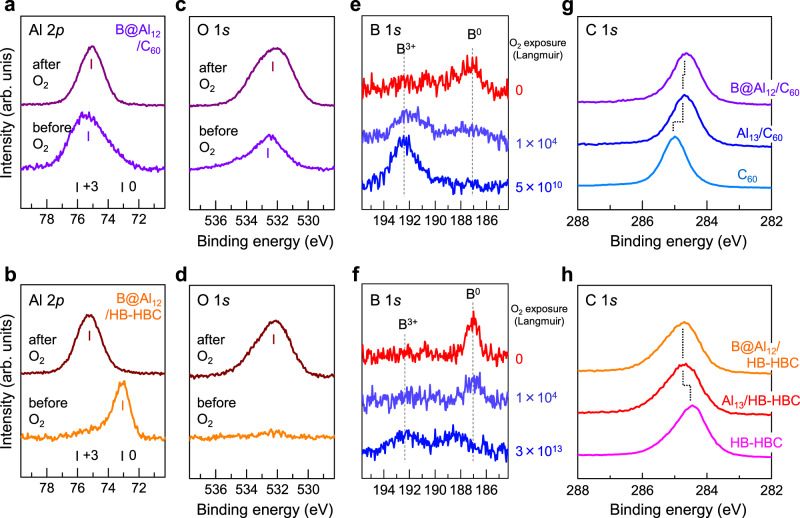
XPS spectra for B@Al_12_ on the C_60_ and HB-HBC substrates. **a**–**d** XPS spectra around the core Al 2*p* levels for **a** B@Al_12_ on C_60_ and **b** B@Al_12_ on HB-HBC before (lower) and after (upper) O_2_ exposure (at 5 × 10^10^ L), along with **c**, **d** O 1*s* for each state. **e**, **f** XPS spectra around the B 1*s* core levels for **e** B@Al_12_ on C_60_ **f** B@Al_12_ on HB-HBC before (top) and after (lower) O_2_ exposure. **g**, **h** XPS spectra around the C 1*s* core levels of the underlying **g** C_60_ or **h** HB-HBC are also shown for the depositions of B@Al_12_ and Al_13_. The reference binding energies (BEs) of Al 2*p* and B 1*s* for the bulk Al and B (Al^0^/B^0^ and Al^3+^/B^3+^) and O 1*s* (O^2−^) are marked by vertical bars. The BEs for Al 2*p* show the presence of zerovalent Al^0^ only for Al_13_ on HB-HBC before O_2_ exposure, while the other BEs are in the vicinity of Al^3+^, indicating the presence of oxidized Al atoms. After the deposition of 0.6 ML B@Al_12_ (violet) and Al_13_ (blue) on C_60_, the C 1*s* peak in (**g**) shifts by ~0.3 eV toward a lower BE from that before deposition (light blue), showing an anionic C_60_^−^ state. After the deposition of 0.6 ML B@Al_12_ (orange) and Al_13_ (red) on HB-HBC, the C 1*s* peak in (**h**) shifts by ~0.25 eV toward a higher BE than that before deposition (pink), showing a cationic HB-HBC^+^ state. Importantly, with B atom doping, B@Al_12_ is stabilized even in the cationic form, as shown by the tailing peak of Al 2*p* in (**a**) and the non-oxidized B^0^ component in (**e**).

Figure 4a, b show the XPS spectra around the Al 2*p* core levels for the B@Al_12_ deposited on C_60_ and the B@Al_12_ deposited on HB-HBC, respectively, before (lower) and after (upper) O_2_ exposures, similar to the spectra in Fig. 1. These XPS spectra show that Al atoms on C_60_ are substantially oxidized without O_2_ exposure, but the tailing peak in the Al^0^ region implies that some Al atoms survive without oxidation. In contrast, the Al atoms of B@Al_12_ deposited on HB-HBC are not oxidized in the same manner as those of Al_13_, as shown in Fig. 1b.

Upon O_2_ exposure, the Al 2*p* XPS peak for the Al atoms deposited on C_60_ becomes shaper upon oxidation, while the Al atoms on HB-HBC are sequentially oxidized to Al^3+^. As shown in Fig. 4c, d, the corresponding O 1*s* component can be observed in the lower trace of Fig. 4c even without O_2_ exposure, but the peak intensity is lower than that observed for the Al_13_ on C_60_ (see Fig. 1c). In fact, the intensity of the O 1*s* peak increases with O_2_ exposure, as shown in Fig. 4c. These results show that the B@Al_12_ NCs on C_60_ are reactive, but that the oxidation rate is surpressed because of the geometrical stabilization induced by B atom encapsulation.

As shown in Fig. 4e, f, the B 1*s* XPS spectra show the effect of such B atom encapsulation. More specifically, despite a similar oxidative reactivity between the Al and B atoms^55,56^, the B 1 s peak for the B@Al_12_ on C_60_ shows that a non-oxidized B^0^ component can be observed even for the nascent B@Al_12_ on C_60_, showing that the oxidation of B atoms to achieve the B^3+^ state is significantly slower than the corresponding oxidation of Al atoms under O_2_ exposure. More importantly, the B 1*s* peak for the B@Al_12_ on HB-HBC can be observed at an O_2_ exposure amount up to ~1 × 10^4^ L, at which point the majority of Al atoms are oxidized. Furthermore, Fig. 4g, h show the C 1*s* XPS spectra of B@Al_12_ on the C_60_ and HB-HBC substrates, respectively, wherein a behavior similar to that of Al_13_ deposition can be observed. More specifically, for the B@Al_12_ on C_60_, the C 1*s* peak (Fig. 4g) shifts toward a lower BE by ~0.25 eV, while for the B@Al_12_ on HB-HBC, the C 1*s* peak (Fig. 4h) shifts toward a higher BE by ~0.25 eV, suggesting the formation of a B@Al_12_^−^/HB-HBC^+^ CT complex.

When the oxidation rate of B@Al_12_ is similarly evaluated based on the peak area ratio of the non-oxidized component (${{{{{{\rm{S}}}}}}}_{A{l}^{0}}$) to the oxidized component (${{{{{{\rm{S}}}}}}}_{A{l}^{3+}}$) (see Supplementary Fig. 9), the *O*_B@Al12_ value is the same with the *O*_Al13_ value within experimental uncertainties, resulting in similar *V*_B@Al12_(O_2_) and *V*_Al13_(O_2_) values, as plotted in Fig. 3. Upon B atom encapsulation, all Al atoms become surface Al atoms of the Al_12_ cage, while in contrast, Al_13_ consists of twelve surface Al atoms and one central Al atom. The same oxidative rates observed for B@Al_12_ and Al_13_ therefore indicate that B@Al_12_ is more robust because these equivalent rates were obtained despite the contribution of the central Al atom of Al_13_.

### Theoretical calculations on the charge distributions for the 13-mer anions and cations

**Fig. 5:**
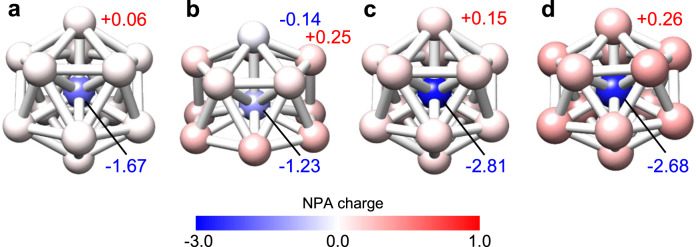
Calculated NPA charge distributions for Al_13_ and B@Al_12_. **a**–**d** Natural population analysis (NPA) distributions for **a** Al_13_^−^, **b** Al_13_^+^, **c** B@Al_12_^−^, and **d** B@Al_12_^+^ for the optimized structures using PBE0 with 6-311+G(d) for Al_13_^−^ and B@Al_12_^−^ or with 6-311G(d) for Al_13_^+^ and B@Al_12_^+^. Along with the representative values, the charge amount is expressed by the color gradation: positive in red and negative in blue. The icosahedral *I*_h_ symmetries for Al_13_^−^ and B@Al_12_^−^ are lowered to *C*_1_ for Al_13_^+^ and *C*_i_ for B@Al_12_^+^ owing to electron deficiency in the 2P shell. In general, a central Al/B atom is negatively charged, while the surface Al atoms are positively charged, with the exception of a top Al atom in the distorted Al_13_^+^ in (**b**).

For Al_13_^−^, B@Al_12_^−^, Al_13_^+^, and B@Al_12_^+^, although theoretical calculations have been reported by several groups^36,38,57,58^, density functional theory (DFT) calculations are collectively performed to explain the different oxidation behaviors observed for Al_13_/B@Al_12_ on the C_60_ and HB-HBC substrates. The results are presented in Fig. 5, and the Cartesian coordinates are summarized in Supplementary Table 4. For the equilibrium structures, the averaged Al–Al bond lengths are 0.2794 nm for icosahedral Al_13_^−^ and 0.2675 nm for icosahedral B@Al_12_^−^. The shortened Al–Al bond in B@Al_12_^−^ is ascribed to relaxed geometric strains due to the presence of a small central B atom inside the Al_12_ cage. For both Al_13_^+^ and B@Al_12_^+^, the structural symmetry is lowered, giving *C*_1_ symmetry for Al_13_^+^ and *C*_i_ symmetry for B@Al_12_^+^, and this was attributed to the electron deficiency of 2P shell closure.

In terms of the charge distributions of Al_13_^−^/B@Al_12_^−^ and Al_13_^+^/B@Al_12_^+^, natural population analysis (NPA) shows that the central Al/B atom is negatively charged, while the surface Al atoms (*ρ* (Al)) have a positive charge of +0.06/+0.15 for Al_13_^−^/B@Al_12_^−^. Compared to the central Al atom (*ρ* (Al) = −1.67), the central B atom is more negatively charged (*ρ* (B) = −2.68), and the surrounding twelve Al atoms (*ρ* (Al) = +0.15) are more positively charged than those in Al_13_^−^ (*ρ* (Al) = +0.06). For Al_13_^+^/B@Al_12_^+^, the positive charges are delocalized over all Al atoms in the cluster, with the exception of one negatively charged Al atom. Therefore, these theoretical calculations show common electronic features wherein the negatively charged central atoms are masked by the surrounding Al atoms.

## Discussion

### Charge state of the Al_13_/B@Al_12_ deposited on a *p*-type substrate and reaction mechanism

The charge states of the deposited Al_13_/B@Al_12_ SAs were found to be significantly influenced by the cluster–surface interactions, which in turn are affected by the molecular character of the organic substrate. More specifically, the *p*-type organic substrate of HB-HBC was found to electronically stabilize the halogen-like Al_13_/B@Al_12_ NCs on the surface by donating an electron, which led to electron shell closure.

The ultraviolet photoelectron spectroscopy (UPS) reveals the electronic states of the organic substrates (see the UPS spectrum for HB-HBC in Supplementary Fig. 10). More specifically, before the deposition of Al_*n*_, the HOMO energies of the C_60_ and HB-HBC are 2.3 eV^59^ and 1.6 eV (at the peak maximum) below the Fermi level (*E*_F_), respectively. In addition, the LUMO levels can be accessed by two-photon photoemission spectroscopy; LUMO energies of C_60_ and HB-HBC are 0.7 eV^59^ and 1.4 eV above the *E*_F_ for C_60_ and HB-HBC, respectively (see Supplementary Fig. 11). These HOMO and LUMO energies indicate that C_60_ (HB-HBC) can be regarded as *n*-type (*p*-type) substrates, wherein C_60_ accepts an electron to its LUMO, while HB-HBC donates an electron from its HOMO. Indeed, the quantitative evaluations carried out for the energetics of CT complexation between Al_13_/B@Al_12_ and C_60_/HB-HBC reasonably explain the formation of Al_13_^+^C_60_^−^/B@Al_12_^+^C_60_^−^ and Al_13_^−^HB-HBC^+^/B@Al_12_^−^HB-HBC^+^ (see Supplementary Note 5 and related contents, i.e., Supplementary Fig. 12, Supplementary Table 2, and Supplementary Table 3).

In the context of O_2_ chemisorption on the surfaces, the adsorbed O_2_ molecules with two unpaired electrons accept an electron from the surface, forming superoxide (O_2_^−^) or peroxide (O_2_^2−^) ions^60,61^. Furthermore, the adsorption energy of two O atoms is larger than the dissociation energy of a single O_2_ molecule^62^, and therefore, O atoms are preferentially bound to the surface via a dissociative electron attachment process^61^. When O_2_ molecules react with CT complexes on a substrate, the O_2_ molecules preferentially attack the electron-rich sites of the anions. At the deposition of Al_13_^−^/B@Al_12_^−^ SAs onto the C_60_ and HB-HBC substrates, as mentioned above, an electron transfer takes place to form Al_13_^+^C_60_^−^/B@Al_12_^+^C_60_^−^ on C_60_ and Al_13_^−^HB-HBC^+^/B@Al_12_^−^HB-HBC^+^ on HB-HBC. Comparing the electron affinities (*EA*s) of the C_60_ (2.68 eV)^63^ and Al_13_/B@Al_12_ SAs (3.1–3.6 eV)^33,64,65^, it is easier to transfer an electron from C_60_^−^ to O_2_ than from Al_13_^−^/B@Al_12_^−^. In other words, C_60_^−^, which is generated by the deposition of Al_13_/B@Al_12_, facilitates the dissociative electron attachment of O_2_, resulting in the immediate oxidation of Al_13_^+^/B@Al_12_^+^ cations with O_2_^−^ or O^−^ through a Coulombic attraction. Therefore, the SA nature of Al_13_^−^/B@Al_12_^−^ is reinforced by *p*-type molecular decoration, which renders it possible to fabricate assembled surfaces of chemically robust Al-based SAs.

To conclude, we have successfully characterized the series of Al_*n*_ NCs deposited on an *n*-type C_60_ and *p*–type HB-HBC substrates. The XPS results reveal that the *n*-type C_60_ substrate possessing a high *EA* withdraws an electron from the Al_*n*_ NCs, resulting in a deviation from the electron shell closure. In contrast, the *p*-type HB-HBC substrate donates one electron to the Al_*n*_ NCs, generating electronically stable Al_13_^−^/B@Al_12_^−^ SAs (40 e^−^). The chemical stabilities of the deposited Al_*n*_ examined by step-by-step O_2_ exposure are shown to be significantly influenced by their charge states on the surface, wherein the stability is enhanced in the 40 e^−^ systems of Al_13_/HB-HBC and B@Al_12_/HB-HBC along with icosahedral rigidity.

Overall, we have demonstrated the importance of optimizing the cluster–surface interactions to achieve stable depositions of Al_13_^−^/B@Al_12_^−^ SAs. It has also been demonstrated that the molecular decoration of a substrate aids in controlling the local electronic state through the generation of such cluster–surface interactions. We believe that this molecular strategy for the stable deposition of Al_13_/B@Al_12_ could facilitate the fabrication of SA assemblies for all functional SAs generated in the gas phase.

## Methods

### Sample preparation

The samples of Al_*n*_ or Al_*n*_B_*m*_ NCs deposited on organic C_60_ and HB-HBC substrates were prepared in an integrated vacuum chamber, including an MSP source, NC deposition, organic evaporation, and photoelectron spectroscopy systems^16,40,41^. The organic C_60_ and HB-HBC substrates were prepared on cleaned HOPG by thermal evaporation in ultrahigh vacuum (UHV) conditions (<3 × 10^−8^ Pa). The thicknesses were controlled at 2 and 5 MLs for C_60_ and HB-HBC, respectively, and were monitored using a quartz crystal microbalance. Commercially available C_60_ (Aldrich, sublimed, 99.9%) was used, while HB-HBC was synthesized (see Supplementary Note 1)^66^.

Anionic Al clusters (Al_*n*_^−^) were generated using an MSP system (Ayabo Corp. nanojima-NAP-01)^25^, in which the Al targets were sputtered with Ar^+^ ions in the MSP aggregation cell. After clustering atomic Al vapors into Al_*n*_^−^ in a cooled (77 K) He gas flow, the Al_*n*_^−^ NCs were introduced into a quadrupole (Q) mass filter (Extrel CMS; MAX-16000) through ion optics. The production conditions were optimized by monitoring the mass spectra of Al_*n*_^−^ (see Supplementary Fig. 2) to maximize the ion intensities at the chosen *m/z* ratios. The mass-selected Al_*n*_^−^ NCs were then deposited on the C_60_ and HB-HBC substrates with a mass resolution of *m*/Δ*m* ~70, which was sufficient to exclude the co-deposition of minor products with neighboring *m/z* values (see Supplementary Fig. 2). The collision energy of the Al_*n*_^−^ ions was controlled by applying a bias voltage to the substrates (typically +5 V), satisfying the soft-landing conditions (<10 eV/cluster). The number of deposited Al_*n*_^−^ ions was counted as 2.9 × 10^13^ clusters, where the coverage of Al_*n*_ on the substrates was estimated as 0.6 MLs, assuming a deposition area of 2.8 × 10^13^ nm^2^ (6 mm in diameter) and an Al_*n*_ size estimated by a cubic-root interpolation between the sizes of the Al atom (*n* = 1) and the icosahedral Al_*n*_ (*n* = 13 and 55) (i.e., 0.62 nm for *n* = 7 and 0.98 nm for *n* = 24 in diameter). The estimated coverage was verified by XPS and UPS measurements with the step-by-step deposition of NCs^40,41^. The deposited samples were transferred to the photoelectron spectroscopy system connected to the cluster deposition system while maintaining UHV conditions. More detailed procedures for sample preparation were described in Supplementary Note 2.

### Photoelectron spectroscopy

XPS measurements were performed using an Mg Kα (*hν* = 1253.6 eV) X-ray source. Photoelectrons emitted from the sample surface were collected with a hemispherical electron energy analyzer (VG SCIENTA, R3000) at a detection angle of 45° from the surface normal. The BE was calibrated using the Au 4 f core level (84.0 eV). It was ensured that no charging effect was observed during any of the XPS measurements. In the XPS analyses, after subtracting the Shirley background, peak fitting was performed by instrumental broadening determined from the Au 4f peak profile (Voigt function with a full width at half maximum (FWHM) of 1.09 eV; the Gaussian and Lorentzian widths were 0.75 and 0.56 eV, respectively). A He–I discharge lamp (*hν* = 21.22 eV) was used for the UPS measurements.

To examine the oxidative reactivities of the deposited Al_*n*_ NCs, the samples were exposed to O_2_. The amount of O_2_ exposure was defined as Langmuir units (L = 1.33 × 10^−4^ Pa·s). The O_2_ gas was introduced into the XPS/UPS system using a variable leak valve for low exposure levels (≤10^4^ L). At higher exposure levels (>10^10^ L), the sample was exposed to O_2_ in a UHV chamber isolated from the XPS/UPS system. All XPS/UPS measurements and O_2_ exposures were performed at room temperature.

### Density functional theory (DFT) calculations

Geometry optimizations for the Al_13_^−^, B@Al_12_^−^, Al_13_^+^, and B@Al_12_^+^ cluster ions with singlet spin states were performed by DFT implemented in the Gaussian 16 program^67^. All equilibrium geometries were optimized until no imaginary frequencies were found. The hybrid exchange-correlation function PBE0^68,69^ was employed at 6-311+G(d) for Al_13_^−^ and B@Al_12_^−^ and at 6-311G(d) for Al_13_^+^ and B@Al_12_^+^. Population analyses were performed using NPA^70^ for the total electron density obtained at the same level of DFT calculations.

## Supplementary information

Supplementary Information

Peer Review File

## Data Availability

The data that support the findings of this study can be found in the manuscript, Supplementary information, or are available from the corresponding author upon request.

## Code availability

The codes used for the analysis in the current study are available from the corresponding author upon request.

## References

[1] Cleveland CL, Landman U (1992). Dynamics of cluster–surface collisions. Science.

[2] Bromann K (1996). Controlled deposition of size-selected silver nanoclusters. Science.

[3] Landman U (2005). Materials by numbers: computations as tools of discovery. Proc. Natl Acad. Sci. USA.

[4] Johnson GE, Gunaratne D, Laskin J (2016). Soft- and reactive landing of ions onto surfaces: concepts and applications. Mass Spectrom. Rev..

[5] Zhu C, Yang G, Li H, Du D, Lin Y (2015). Electrochemical sensors and biosensors based on nanomaterials and nanostructures. Anal. Chem..

[6] Popok VN, Barke I, Campbell EEB, Meiwes-Broer K-H (2011). Cluster–surface interaction: From soft landing to implantation. Surf. Sci. Rep..

[7] Haruta M (2002). Catalysis of gold nanoparticles deposited on metal oxides. Cattech.

[8] Yoon B (2005). Charging effects on bonding and catalyzed oxidation of CO on Au_8_ clusters on MgO. Science.

[9] Landman U, Yoon B, Zhang C, Heiz U, Arenz M (2007). Factors in gold nanocatalysis: oxidation of CO in the non-scalable size regime. Top. Catal..

[10] Crampton AS, Rötzer MD, Landman U, Heiz U (2017). Can support acidity predict sub-nanometer catalyst activity trends?. ACS Catal..

[11] Marcus, R. A. Electron transfer reactions in chemistry: theory and experiment (Nobel lecture). *Angew. Chem. Int. Ed*. **32**, 1111–1121 (1993).

[12] Wang Y (1992). Photophysical properties of fullerenes/N,N-diethylanaline charge transfer complexes. J. Phys. Chem..

[13] Akamatsu H, Inokuchi H, Matsunaga Y (1954). Electrical conductivity of the perylene–bromine complex. Nature.

[14] Yamada J, Akutsu H, Nishikawa H, Kikuchi K (2004). New trends in the synthesis of π-electron donors for molecular conductors and superconductors. Chem. Rev..

[15] Duffe S (2010). Penetration of thin C_60_ films by metal nanoparticles. Nat. Nanotechnol..

[16] Nakaya M, Iwasa T, Tsunoyama H, Eguchi T, Nakajima A (2014). Formation of a superatom monolayer using gas-phase-synthesized Ta@Si_16_ nanocluster ions. Nanoscale.

[17] Castleman AW, Khanna SN (2009). Clusters, superatoms, and building blocks of new materials. J. Phys. Chem. C..

[18] Castleman AW (2011). From elements to clusters: the periodic table revisited. J. Phys. Chem. Lett..

[19] Jena P (2013). Beyond the periodic table of elements: The role of superatoms. J. Phys. Chem. Lett..

[20] Tomalia DA, Khanna SN (2016). A Systematic framework and nanoperiodic concept for unifying nanoscience: Hard/soft nanoelements, superatoms, meta-atoms, New emerging properties, periodic property patterns, and predictive Mendeleev-like nanoperiodic tables. Chem. Rev..

[21] Luo Z, Castleman AW (2014). Special and general superatoms. Acc. Chem. Res..

[22] Reber AC, Khanna SN (2017). Superatoms: electronic and geometric effects on reactivity. Acc. Chem. Res..

[23] Jena P, Sun Q (2018). Super atomic clusters: design rules and potential for building blocks of materials. Chem. Rev..

[24] Ferrari P, Vanbuel J, Janssens E, Lievens P (2018). Tuning the reactivity of small metal clusters by heteroatom doping. Acc. Chem. Res..

[25] Tsunoyama H, Shibuta M, Nakaya M, Eguchi T, Nakajima A (2018). Synthesis and characterization of metal-encapsulating Si_16_ cage superatoms. Acc. Chem. Res.

[26] Bergeron DE, Castleman AW, Morisato T, Khanna SN (2004). Formation of Al_13_I^−^: evidence for the superhalogen character of Al_13_. Science.

[27] Reveles JU, Khanna SN, Roach PJ, Castleman AW (2006). Multiple valence superatoms. Proc. Natl Acad. Sci. USA.

[28] Reber AC, Khanna SN, Castleman AW (2007). Superatom compounds, clusters, and assemblies: ultra alkali motifs and architectures. J. Am. Chem. Soc..

[29] Pal R (2008). Probing the electronic and structural properties of doped aluminum clusters: M Al_12_^−^ (M=Li, Cu, and Au). J. Chem. Phys..

[30] Leuchtner RE, Harms AC, Castleman AW (1991). Aluminum cluster reactions. J. Chem. Phys..

[31] Yin B, Luo Z (2019). Thirteen-atom metal clusters for genetic materials. Coord. Chem. Rev..

[32] Li X, Wu H, Wang X-B, Wang L-S (1998). s-p Hybridization and electron shell structures in aluminum clusters: a photoelectron spectroscopy study. Phys. Rev. Lett..

[33] Knight WD (1984). Electronic shell structure and abundances of sodium clusters. Phys. Rev. Lett..

[34] Khanna SN, Jena P (1992). Assembling crystals from clusters. Phys. Rev. Lett..

[35] Claridge SA (2009). Cluster-assembled materials. ACS Nano.

[36] Yin B, Luo Z (2021). Coinage metal clusters: from superatom chemistry to genetic materials. Coord. Chem. Rev..

[37] Nakajima A, Kishi T, Sugioka T, Kaya K (1991). Electronic and geometric structures of aluminum-boron negative cluster ions (Al_*n*_B_*m*_^*−*^). Chem. Phys. Lett..

[38] Akutsu M (2006). Experimental and theoretical characterization of aluminum-based binary superatoms of Al_12_X and their cluster salts. J. Phys. Chem. A.

[39] Bianconi A, Bachrach RZ, Hagstrom SBM, Flodström SA (1973). Al-A1_2_O_3_ interface study using surface soft-x-ray absorption and photoemission spectroscopy. Phys. Rev. B.

[40] Shibuta M (2015). Chemical characterization of an alkali-like superatom consisting of a Ta-encapsulating Si_16_ cage. J. Am. Chem. Soc..

[41] Ohta T, Shibuta M, Tsunoyama H, Eguchi T, Nakajima A (2016). Charge transfer complexation of Ta-encapsulating Ta@Si_16_ superatom with C_60_. J. Phys. Chem. C..

[42] Walter M (2019). Experimental and theoretical 2*p* core-level spectra of size-selected gas-phase aluminum and silicon cluster cations: chemical shifts, geometric structure, and coordination-dependent screening. Phys. Chem. Chem. Phys..

[43] Kambe T, Haruta N, Imaoka T, Yamamoto K (2017). Solution-phase synthesis of Al_13_^−^ using a dendrimer template. Nat. Commun..

[44] Martinson CWB, Flodstrom SA (1979). Oxygen adsorption on aluminum single crystal faces studied by AES, XPS and LEED. Surf. Sci..

[45] van Heijnsbergen D, Demyk K, Duncan MA, Meijer G, von Helden G (2003). Structure determination of gas phase aluminum oxide clusters. Phys. Chem. Chem. Phys..

[46] Burgert R (2008). Spin conservation accounts for aluminum cluster anion reactivity pattern with O_2_. Science.

[47] Sweeny BC (2019). Thermal kinetics of Al_*n*_^−^ + O_2_ (*n* = 2−30): measurable reactivity of Al_13_^−^. J. Phys. Chem. A.

[48] Sweeny BC (2020). Redefining the mechanism of O_2_ etching of Al_*n*_^−^ superatoms: an early barrier controls reactivity, analogous to surface oxidation. J. Phys. Chem. Lett..

[49] Nakajima A, Hoshino K, Naganuma T, Sone Y, Kaya K (1991). Ionization potentials of aluminum-sodium bimetallic clusters (Al_*n*_Na_*m*_). J. Chem. Phys..

[50] Aguado A, Lόpez JM (2009). Structures and stabilities of Al_*n*_^+^, Al_*n*_, and Al_*n*_^−^ (*n* = 13-34) clusters. J. Chem. Phys..

[51] Drebov N, Ahlrichs R (2010). Structures of Al_*n*_, its anions and cations up to *n* = 34: a theoretical investigation. J. Chem. Phys..

[52] Iwasa T, Nakajima A (2013). Geometric, electronic, and optical properties of a boron-doped aluminum cluster of B_2_Al_21_^−^. Chem. Phys. Lett..

[53] Smith JC, Reber AC, Khanna SN, Castleman AW (2014). Boron substitution in aluminum cluster anions: magic clusters and reactivity with oxygen. J. Phys. Chem. A.

[54] Chauhan V, Reber AC, Khanna SN (2018). Strong lowering of ionization energy of metallic clusters by organic ligands without changing shell filling. Nat. Commun..

[55] Bauer SH (1996). Oxidation of B, BH, BH_2_, and B_*m*_H_*n*_ species: thermochemistry and kinetics. Chem. Rev..

[56] Garland NL, Nelson HH (1992). Temperature dependence of the kinetics of the reaction of Al+O_2_→AlO+O. Chem. Phys. Lett..

[57] Gong XG, Kumar V (1993). Enhanced stability of magic clusters: A case study of icosahedral Al_12_X, A=B, Al, Ga, C, Si, Ge, Ti, As. Phys. Rev. Lett..

[58] Zhao J, Du Q, Zhou S, Kumar V (2020). Endohedrally doped cage clusters. Chem. Rev..

[59] Shibuta M (2016). Direct observation of photocarrier electron dynamics in C_60_ films on graphite by time-resolved two-photon photoemission. Sci. Rep..

[60] Höfer U, Morgen P, Wurth W, Umbach E (1985). Metastable molecular precursor for the dissociative adsorption of oxygen on Si(111). Phys. Rev. Lett..

[61] Libisch F, Huang C, Liao P, Pavone M, Carter EA (2012). Origin of the energy barrier to chemical reactions of O_2_ on Al(111): Evidence for charge transfer, not spin selection. Phys. Rev. Lett..

[62] Toyoshima I, Somorjai GA (1979). Heats of chemisorption of O_2_, H_2_, CO, CO_2_, and N_2_ on polycrystalline and single crystal transition metal surfaces. Catal. Rev. Sci. Eng..

[63] Huang DL, Dau PD, Liu HT, Wang LS (2014). High-resolution photoelectron imaging of cold C_60_^−^ anions and accurate determination of the electron affinity of C_60_. J. Chem. Phys..

[64] Ganteför G, Gausa M, Meiwes-Broer KH, Lutz HO (1988). Photoelectron spectroscopy of jet-cooled aluminium cluster anions. Z. Phys. D..

[65] Kawamata H, Negishi Y, Nakajima A, Kaya K (2001). Electronic properties of substituted aluminum clusters by boron and carbon atoms (Al_*n*_B_*m*_^−^/ Al_*n*_C_*m*_^−^); New insights into s-p hybridization and perturbed shell structures. Chem. Phys. Lett..

[66] Rathore R, Burns CL (2003). A practical one-pot synthesis of soluble hexa-*peri*-hexabenzocoronene and isolation of its cation-radical salt. J. Org. Chem..

[67] Frisch, M. J. et al. Gaussian 16 Revision A.03 (Gaussian, Wallingford, CT, USA, 2016).

[68] Adamo C, Barone V (1999). Toward reliable density functional methods without adjustable parameters: The PBE0 model. J. Chem. Phys..

[69] Perdew JP, Burke K, Ernzerhof M (1996). Generalized gradient approximation made simple. Phys. Rev. Lett..

[70] Reed AE, Weinstock RB, Weinhold F (1985). Natural population analysis. J. Chem. Phys..

