# Supplementary material for: Al13− and B@Al12− superatoms on a molecularly decorated substrate
Source: Nat Commun. 2022 Mar 14;13:1336. doi: 10.1038/s41467-022-29034-9 (PMC8921336; doi:10.1038/s41467-022-29034-9)
Supplement: Supplementary file 1 — Supplementary Information [file 41467_2022_29034_MOESM1_ESM.pdf]

Supplementary Information for

Al_{13}^{-} and $B@Al_{12}^{-}$ superatoms on a molecularly decorated substrate

Masahiro Shibuta[1], Tomoya Inoue[2], Toshiaki Kamoshida[2], Toyoaki Eguchi[3], and Atsushi Nakajima[1,2,*]

[1] *Keio Institute of Pure and Applied Sciences (KiPAS), Keio University, 3-14-1 Hiyoshi, Kohoku-ku, Yokohama 223-8522, Japan*

[2] *Department of Chemistry, Faculty of Science and Technology, Keio University, 3-14-1 Hiyoshi, Kohoku-ku, Yokohama 223-8522, Japan*

[3] *Department of Physics, Graduate School of Science, Tohoku University, 6-3 Aramaki Aza-Aoba, Aoba-ku, Sendai 980-8578, Japan*

*Address correspondence to A. Nakajima
Tel: +81-45-566-1712, Fax: +81-45-566-1697, E-mail: nakajima@chem.keio.ac.jp

Table of Contents

Supplementary Fig. 1: Molecular structure of HB-HBC. 2

Supplementary Note 1: Synthesis of HB-HBC molecule. 2

Supplementary Note 2: Synthesis and deposition of Al_n and Al_nB_m NCs. 3

Supplementary Fig. 2: Mass spectrum for the Al_n anions (n = 8–50). 4

Supplementary Fig. 3: STM images of $Lu@Si_{16}$ superatoms deposited on the HB-HBC substrate. 5

Supplementary Note 3: Evaluation of peak shifts of C 1*s* for Al_{13}/C_{60} and Al_{13}/HB-HBC. 6

Supplementary Fig. 4: XPS C 1*s* peaks for Al_{13}/C_{60} and Al_{13}/HB-HBC. 7

Supplementary Fig. 5: XPS spectra of Al 2*p* for Al_n (n = 7–24)) on the HB-HBC substrate. 8

Supplementary Fig. 6: Peaks of XPS Al 2*p* for Al_n (n = 7–24) on the HB-HBC substrate. 9

Supplementary Fig. 7: C 1*s* peak positions in the XPS spectra for Al_n on the C_{60} and HB-HBC substrates. 10

Supplementary Note 4: Oxygen exposure amount of $V_{Aln}(O_2)$ required to completely oxidize the Al_n NCs. 11

Supplementary Table 1: Values of the linear slope (O_{Aln}) and the intersection ($V_{Aln}(O_2)$), and the relative reactivity. 11

Supplementary Fig. 8: Mass spectrum of the Al-B mixed nanocluster anions, $Al_nB_m^{-}$ (n = 11–13, m = 0–4). 12

Supplementary Fig. 9: Oxidative behaviors in the Al 2*p* XPS spectra for $B@Al_{12}$ on HB-HBC. 13

Supplementary Fig. 10: UPS spectra ($h\nu$ = 21.22 eV) for HB-HBC on HOPG at a coverage of 2 MLs. 14

Supplementary Fig. 11: 2PPE spectra for HB-HBC on HOPG at a coverage of 2 MLs. 15

Supplementary Note 5: Energetics for charge transfer complexation. 16

Supplementary Fig. 12: Schematic potential curves for the complexation between $B@Al_{12}$ and C_{60}/HB-HBC. 18

Supplementary Table 2: Experimental and calculated ionization energies (E_i) and electron affinities (*EA*). 19

Supplementary Table 3: Calculated endothermic dissociation limits of the cations and anions (ΔE). 20

Supplementary Table 4: Cartesian coordinates for Al_{13}^{-}, $B@Al_{12}^{-}$, Al_{13}^{+}, and $B@Al_{12}^{+}$. 21

Supplementary References 23

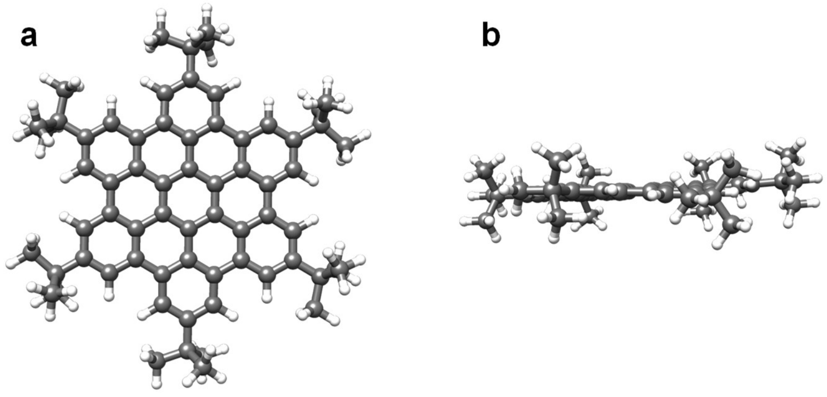

Supplementary Fig. 1 Molecular structure of HB-HBC. a, **b** Molecular structure of hexa-*tert*-butyl-hexa-*peri*-hexabenzocoronene (HB-HBC, $C_{66}H_{66}$): (**a**) top view and (**b**) side view.

Supplementary Note 1 Synthesis of HB-HBC molecule.

The hexa-*tert*-butyl-hexa-*peri*-hexabenzocoronene (HB-HBC, $C_{66}H_{66}$) was synthesized with reference to a previous report by Rathore *et al*[1]. In the synthesis procedures, a nitromethane solution (120 mL) of anhydrous ferric chloride (19.4 g) was slowly added to a solution of hexaphenyl benzene (3.2 g) and *tert*-butyl chloride (5.8 mL) in dichloromethane (200 mL) under argon gas bubbling, keeping the solution temperature at 295 K. The dark red solution was stirred for 3 h at 313 K and was poured into methanol, forming a yellow precipitate of HB-HBC molecules. The HB-HBC precipitate was washed by cold methanol repeatedly. The crude precipitate was then filtered to obtain pure HB-HBC powder.

Supplementary Note 2 Synthesis and deposition of Al_n and Al_nB_m NCs.

The mass-selected Al_n^- or $Al_nB_m^-$ NCs were deposited on organic substrates, in which C_{60} and HB-HBC molecules were pre-decorated prior to the NCs deposition[2,3,4]. A substrate of highly oriented pyrolytic graphite (HOPG) was cleaved in air and was heated at ~700 K for 50 h in an ultrahigh vacuum (UHV) condition (<3×10^{-8} Pa) to remove surface impurities. The C_{60} (Sigma Aldrich, sublimed, 99.9%) and HB-HBC (see Supplementary Note 1) powders separately loaded in quartz effusion cells were degassed with heating in the UHV system before evaporations. During the evaporations, the thickness of C_{60} and HB-HBC was monitored by a quartz microbalance (INFICON, IC5) to obtain the amounts of 2 ML (C_{60}) and 5 ML (HB-HBC), respectively.

Al_n or Al_nB_m NCs were generated in the MSP system (Ayabo Corp. nanojima-NAP-01),[25] in which the pure Al or mixed Al-B targets (Rare Metallic. Co., LTD.) were sputtered with Ar^+ ions in the aggregation cell filled with a cooled (77 K) He buffer gas. Formed Al_n (or Al_nB_m) NC ions were guided through a radio frequency octupole ion guide to an ion bender for charge selection. The negatively charged species (i.e. Al_n^- or $Al_nB_m^-$ NC ions) bended were introduced into a quadrupole mass filter (Extrel CMS; MAX-16000). Since the production of $Al_n^-/Al_nB_m^-$ NCs sensitively depends on the conditions of DC power to the MSP and the flow rates of sputtering Ar and buffer He gases, they were optimized to maximize the ion intensities at the targeted m/z ratios by monitoring the mass spectra of $Al_n^-/Al_nB_m^-$ NCs (see Supplementary Figs. 2 and 8).

The mass-selected Al_n^- (or $Al_nB_m^-$) NCs were deposited on the prepared C_{60} and HB-HBC substrates with the mass resolution of $m/\Delta m$ ~70 during the deposition which was enough to exclude neighboring minor products (see Supplementary Figs. 2 and 8). To achieve a "soft-landing condition" in the NC deposition, a positive bias voltage of +5 V was applied to the substrates to avoid decomposing NCs at the surface deposition. The number of deposited Al_n^- (or $Al_nB_m^-$) ions was counted through an ion current, in which a typical ion current of 300 pA (~1.9×10^9 NC ions per second) was generated. The sample temperature during the deposition was kept at 300 K.

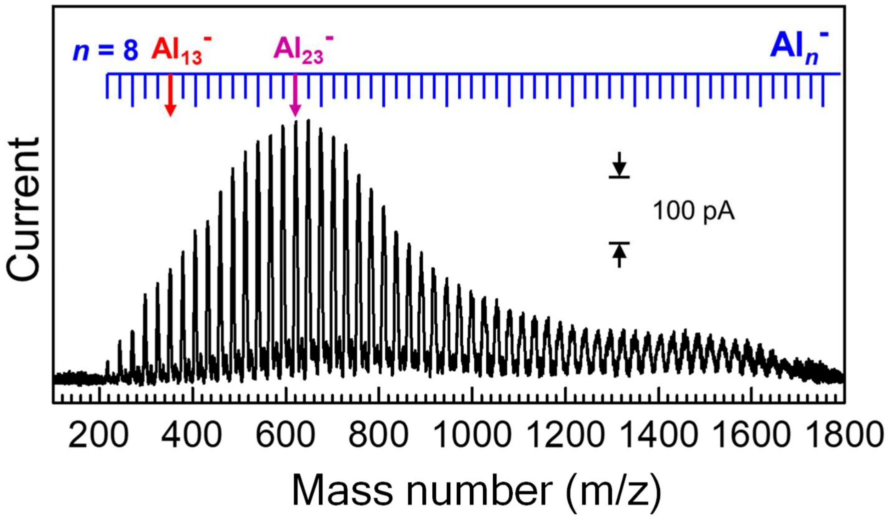

Supplementary Fig. 2 Mass spectrum for the Al_n anions (n = 8–50). With an Al disk target, anionic Al_n^- nanoclusters (NCs) were formed by magnetron sputtering, and the mass distributions were measured using a quadrupole mass spectrometer. Al_n^- NCs are formed with ion currents of a few hundred picoamperes (pA) for n = 7–25, and a specific size of Al_n^- was mass-selectively deposited on a molecularly decorated substrate.

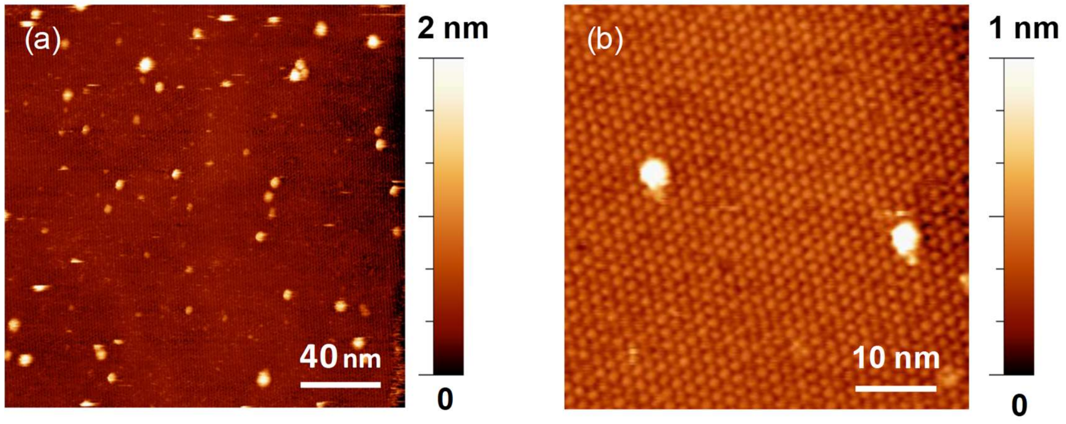

Supplementary Fig. 3 STM images of Lu@Si_{16} superatoms deposited on the HB-HBC substrate. (**a**) wide (200 × 200 nm^2) and (**b**) molecular scale (50 × 50 nm^2) STM images of Lu@Si_{16} (0.3 MLs) on the HB-HBC (5 MLs)/HOPG substrate (3 mm off-centered from the nanocluster deposition beam with 6 mm diameter). Well-aligned protrusions due to the HB-HBC substrate with hexagonal lattice patterns ($a = b =$ 1.79 nm) are observed in (**b**), where the lattice constant is consistent with the in-plane size of the HB-HBC molecule (Supplementary Fig. 1). This means that the HB-HBC film is grown in a flat conformation on the HOPG substrate. Bright dots correspond to the deposited Lu@Si_{16}, showing the successful immobilization of nanoclusters in a monodisperse manner. Note that Lu@Si_{16} (67 e^- valence electrons) is a halogen-like superatom,[5,6] as is Al_{13}, owing to the lack of a single electron to close the electron shell. The imaging conditions of the tip voltage and the tunneling current are −2.0 V and 0.2 pA for (**a**), and −2.0 V and 0.5 pA for (**b**), respectively.

Supplementary Note 3 Evaluation of peak shifts of C 1*s* for Al_{13}/C_{60} and Al_{13}/HB-HBC.

To discuss the XPS results more quantitatively, peak deconvolutions were performed as follows: (1) The C 1*s* peak of the highly oriented pyrolytic graphite (HOPG), which was used as a base substrate and exhibits an asymmetric structure, is fitted with a Doniach−Šunjić line shape[7], and its line shape and energy are fixed as "G." (2) The C 1*s* peak of C_{60} or HB-HBC is fitted by the "G" component and an additional peak component of C_{60} ("F") or HB-HBC ("H"), where the fitting parameters for "F" and "H" are given by the Voigt function. (3) In the deconvolution of the C 1*s* peaks for Al_{13}/C_{60} and Al_{13}/HB-HBC, a new peak component, originating from the C atoms in C_{60} ("N") or HB-HBC ("P") that interact with the deposited Al_{13}, is added using the same contribution ratio of "G" to "F" or "H" obtained in (2). The above deconvoluted components are schematically illustrated in Supplementary Fig. 4. The binding energy (BE) of the extracted component "N" is 0.33 eV (284.75 eV) lower than that of "F," while the BE of the extracted component "P" is 0.50 eV (284.70 eV) higher than that of "H".

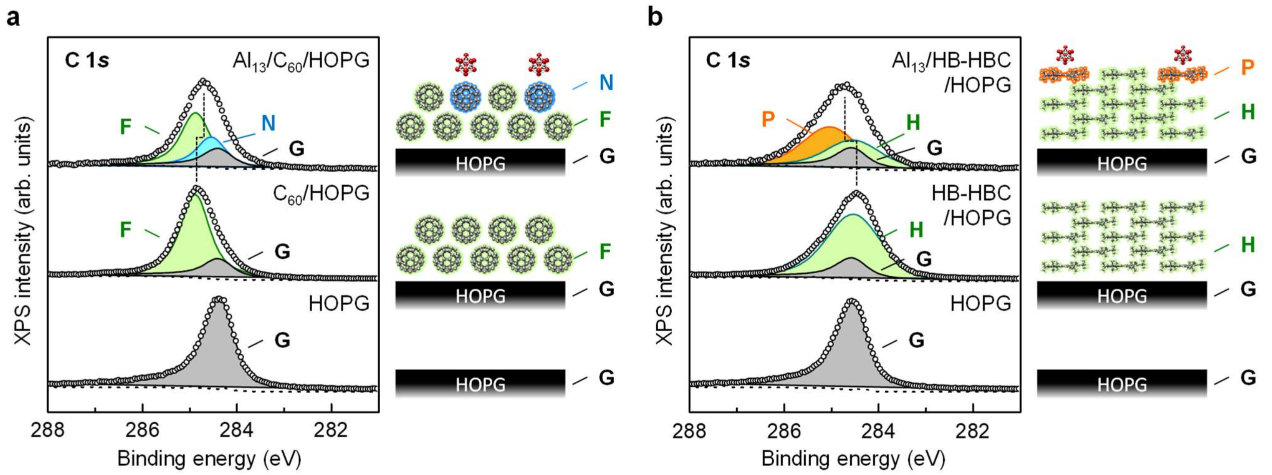

Supplementary Fig. 4 XPS C 1*s* peaks for Al_{13}/C_{60} and Al_{13}/HB-HBC. (a) XPS C 1*s* peak for Al_{13}/C_{60}/HOPG, which is deconvoluted into three peak components for the HOPG base substrate (**G**, gray area), the non-interacted C_{60} (**F**, green area), and the interacted C_{60} with Al_n oxide (**N,** blue area); the binding energy (BE) of the interacted C_{60} peak is 0.33 eV lower than that of the non-interacted C_{60}; the shift amount of 0.33 eV corresponds to the formation of C_{60}^{-} as described in the literature[8]. (**b**) XPS C 1*s* peak for Al_{13}/HB-HBC/HOPG, which is also deconvoluted into three peak components for HOPG (**G**), the non-interacted HB-HBC (**H**, green area), and the interacted HB-HBC with Al_{13} (**P**, orange area); the BE of the interacted HB-HBC peak is 0.50 eV higher than that of the non-interacted HB-HBC. The shift corresponds to the formation of a cationic HB-HBC^{+} state, suggesting that Al_{13} forms a CT complex, such as Al_{13}^{-}/HB-HBC^{+}.

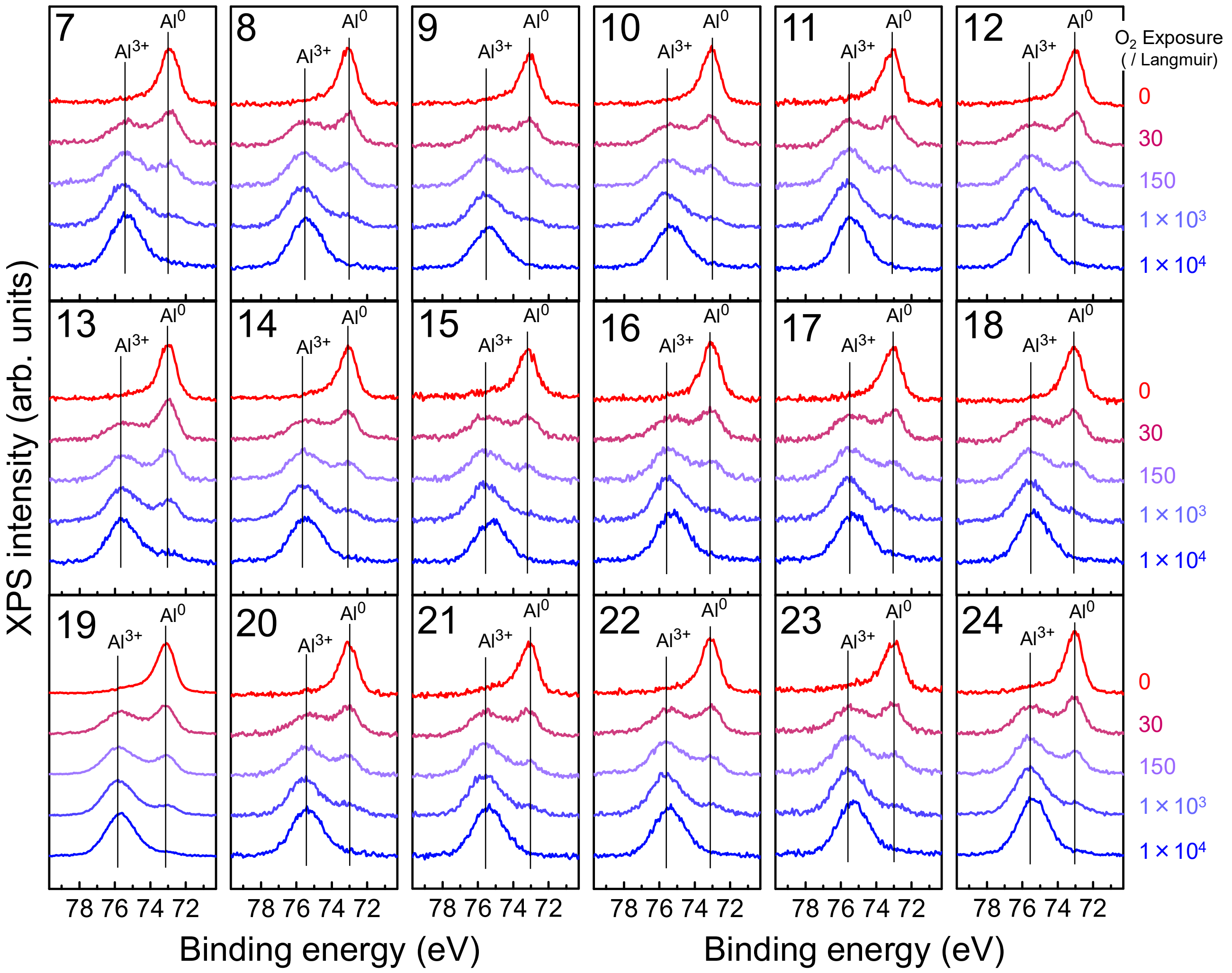

Supplementary Fig. 5 XPS spectra of Al 2*p* for Al_n (*n* = 7–24)) on the HB-HBC substrate. XPS spectra around the core levels of Al 2*p* for the Al_n NCs on HB-HBC at several different O_2 exposures (0– L – 5 × 10^{10} L): 0 L (top), 30 L, 150 L, 1 × 10^3 L, and 1 × 10^4 L (bottom). The peaks are characterized by the two states of Al^0 and Al^{3+}.

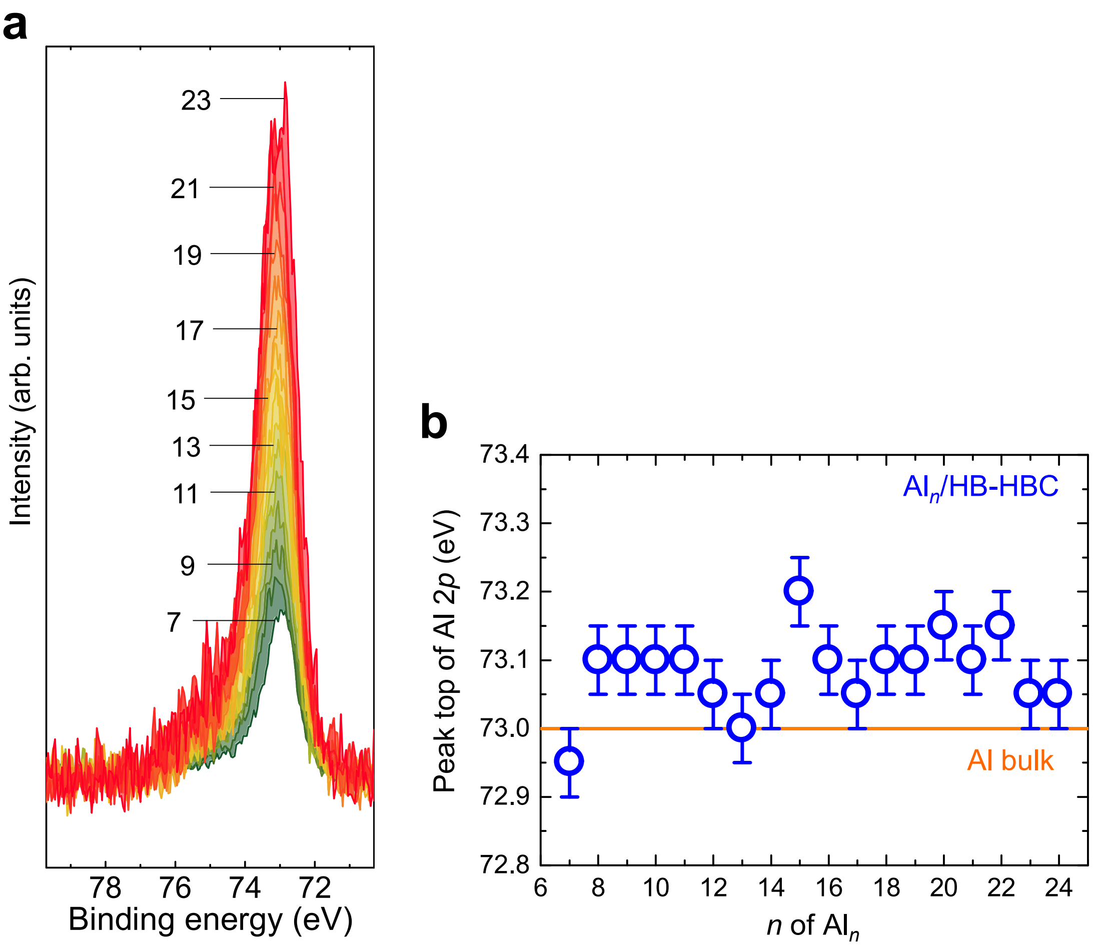

Supplementary Fig. 6 Peaks of XPS Al 2*p* for Al$_n$ (*n* = 7–24) on the HB-HBC substrate.
(a) Superimposed Al 2*p* peaks for Al$_n$ (*n* = 7 – 24) on HB-HBC/HOPG. **(b)** The Al 2*p* peak positions. The peaks appear in the vicinity of zerovalent bulk Al^0 (73.0 eV)[9] with a very slight size dependence (73.0–73.2 eV). Error bars (± 0.05 eV) correspond to the standard error of the mean with an energy step in the XPS measurements.

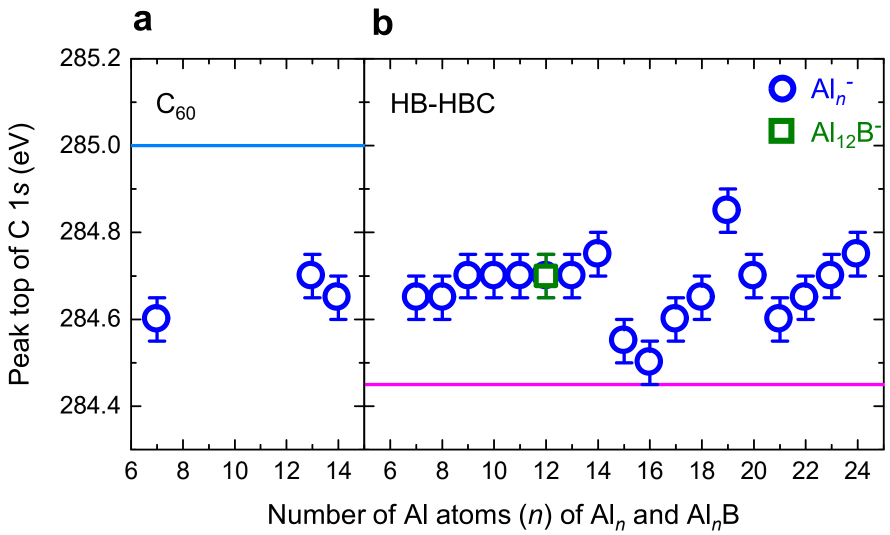

Supplementary Fig. 7 C 1*s* peak positions in the XPS spectra for Al*n* on the C_{60} and HB-HBC substrates. (a, b) Peak positions of C 1*s* for the Al_n (blue open circles) on C_{60} (**a**) and on HB-HBC (**b**) and for the $Al_{12}B$ (green open square) on HB-HBC (**b**). The peaks for both substrates exhibit a shift after the deposition of Al_n; the energy decrease of C 1*s* on C_{60} is −0.3 eV, while and energy increase of C 1*s* on HB-HBC is +0.25 eV with a small size-dependent shift, where the C 1*s* peaks for the C_{60} and HB-HBC substrates originally appear at 285.0 and 284.45 eV, respectively. Error bars (± 0.05 eV) correspond to the standard error of the mean with an energy step in the XPS measurements.

Supplementary Note 4 Oxygen exposure amount of $V_{\mathrm{Al}n}(O_2)$ required to completely oxidize the Al_n NCs.

To evaluate the oxidative reactivity of the Al_n NCs toward O_2, the Al 2*p* XPS peak area ratio of the non-oxidized component to the oxidized component ($R_{\mathrm{Al}n}$) is plotted against the logarithm of the O_2 exposure amount in L ($\log_{10} O_2$) for each size of *n*. The linear slope ($O_{\mathrm{Al}n}$) and the intersection with the x-axis ($V_{\mathrm{Al}n}(O_2)$) are obtained as shown in Supplementary Table 1 below. The $V_{\mathrm{Al}n}(O_2)$ value corresponds to the oxygen exposure amount required to completely oxidize the Al_n NCs. In Supplementary Table 1, the relative reactivity is evaluated by dividing the $V_{\mathrm{Al}n}(O_2)$ value at $n = 13$ by each $V_{\mathrm{Al}n}(O_2)$ value.

Supplementary Table 1 Values of the linear slope ($O_{\mathrm{Al}n}$) and the intersection ($V_{\mathrm{Al}n}(O_2)$), and the relative reactivity normalized at n = 13 for Al_n ($n = 7$–24) and the B@Al_{12} NCs.

n	$O_{\mathrm{Al}n}$	$\log_{10} O_2$	$V_{\mathrm{Al}n}(O_2)$	Relative Reactivity
7	0.322	3.10	1.27×10^3	27.1
8	0.328	3.05	1.12×10^3	30.7
9	0.320	3.13	1.34×10^3	25.8
10	0.316	3.16	1.45×10^3	23.9
11	0.332	3.02	1.04×10^3	33.3
12	0.296	3.37	2.36×10^3	14.6
13	0.220	4.54	3.45×10^4	1.0
14	0.337	2.97	9.29×10^2	37.2
15	0.357	2.80	6.38×10^2	54.1
16	0.339	2.95	8.98×10^2	38.5
17	0.342	2.93	8.46×10^2	40.8
18	0.342	2.92	8.36×10^2	41.3
19	0.316	3.17	1.47×10^3	23.5
20	0.329	3.04	1.09×10^3	31.8
21	0.347	2.88	7.67×10^2	45.0
22	0.370	2.70	5.06×10^2	68.3
23	0.336	2.98	9.45×10^2	36.5
24	0.310	3.22	1.66×10^3	20.8
B@Al_{12}	0.223	4.49	3.11×10^4	1.1

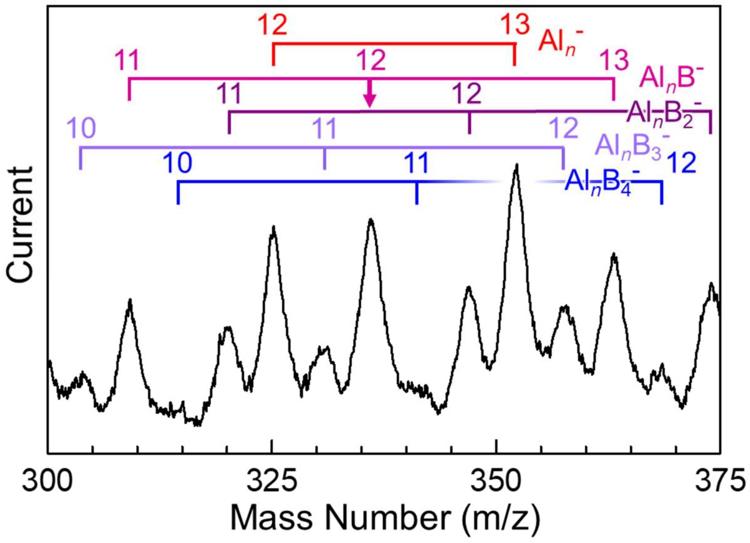

Supplementary Fig. 8 Mass spectrum of the Al-B mixed nanocluster anions, $Al_nB_m^-$ (n = 11–13, m = 0–4). For a B-mixed Al target, $B@Al_{12}^-$ was formed by magnetron sputtering. In addition to pure Al_{12}^- and Al_{13}^-, the B atoms are mixed up to m = 4, and $Al_{12}B_1^-$ can be observed separately from the other composition species in the mass resolution of $m/\Delta m \approx 70$.

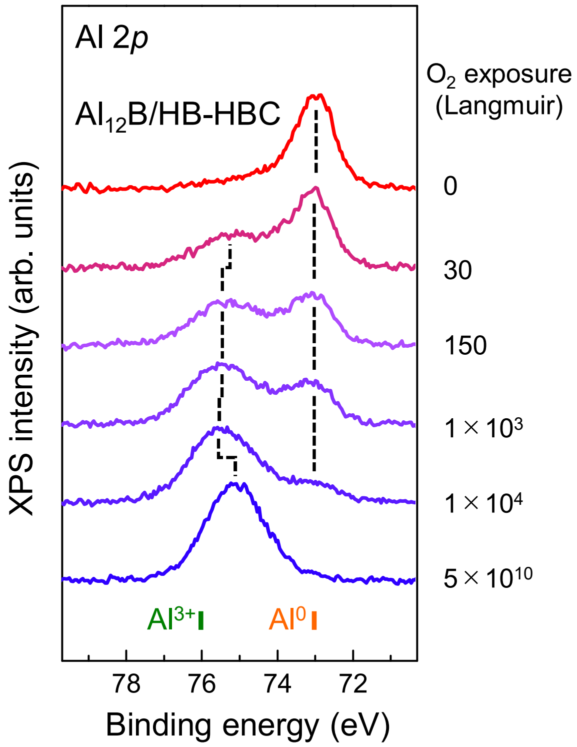

Supplementary Fig. 9 Oxidative behaviors in the Al 2*p* XPS spectra for B@Al_{12} on HB-HBC. With an increasing O_2 exposure, the intensity of the zerovalent component Al^0 peak decreases, while that of the oxidized component Al^{3+} increases accordingly. The oxidative reactivity can be evaluated by the dependence of the O_2 exposure amount from 0 L to 1×10^4 L. At the highest exposure of 5×10^{10} L, the Al 2*p* peak shifts to a lower BE, likely owing to a structural change relevant to the phase transition of aluminum oxide (see the manuscript text for further details).

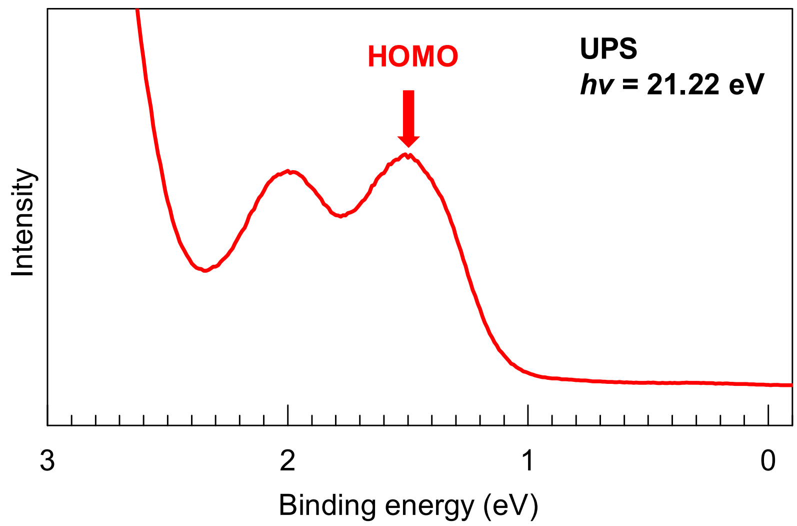

Supplementary Fig. 10 UPS spectra ($h\nu$ = 21.22 eV) for HB-HBC on HOPG at a coverage of 2 MLs. The threshold energy indicated by the solid arrow indicates the HOMO level in the HB-HBC film (i.e., 1.5 eV). Together with the Fermi energy of 4.4 eV, the HOMO is located at 5.9 eV below the vacuum level.

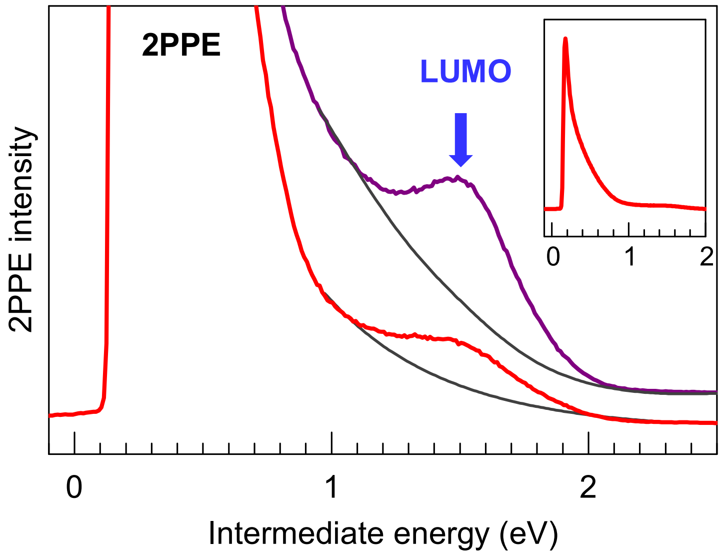

Supplementary Fig. 11 2PPE spectra for HB-HBC on HOPG at a coverage of 2 MLs. The $h\nu$ values are 4.23 eV (bottom, red) and 4.77 eV (top, purple). The horizontal axis is an intermediate energy relative to the Fermi level. Regardless of the $h\nu$ value employed, a peak structure exhibiting a common threshold energy (solid arrow) of 1.5 eV appears, suggesting that the spectral features originate from an unoccupied state. Since no spectral feature is observed at energies lower than 1.5 eV, with the exception of a huge amount of secondary electron signals (see inset), the peak structure can be assigned to the LUMO of HB-HBC. Together with a Fermi energy of 4.4 eV, the LUMO is located at 2.9 eV (4.4–1.5 eV) below the vacuum level.

Supplementary Note 5 Energetics for charge transfer complexation.

For the molecular complexation between Al_{13}/B@Al_{12} and organic molecules, the energy balance toward a charge transfer (CT) is evaluated from the ionization energies (E_i) and the electron affinities (EA) of Al_{13}/B@Al_{12}, C_{60}, and HB-HBC. Using these values, the endothermic dissociation limits of the corresponding cations and anions, ΔE, can be calculated when one electron is transferred between neutral Al_{13}/B@Al_{12} and organic molecules[10], as shown schematically in Supplementary Fig. 12.

The experimental EA of Al_{13}/B@Al_{12} has been previously measured using anion photoelectron spectroscopy in the gas phase[11,12]. However, no accurate experimental E_i for B@Al_{12} has been reported, with only a rough estimation being available[13]; the E_i for B@Al_{12} is less than 7.90 eV (157 nm, F_2 laser) and more than 6.42 eV (193 nm, ArF laser). In contrast, both the EA and E_i values have been theoretically obtained using DFT calculations[14–17]. Although there are some discrepancies between the experimental and calculated values, the calculated E_i and experimental EA were adopted for the estimation for Al_{13}/B@Al_{12}. These values are tabulated in Supplementary Table 2.

The experimentally determined values of E_i and EA of C_{60} have been reported to 7.57 eV[18] and 2.683 eV[19], respectively. However, the corresponding values of HB-HBC molecules have not been reported experimentally or theoretically. Thus, they were evaluated from the HOMO and LUMO energy levels, which were experimentally observed from ultraviolet photoelectron spectroscopy (UPS) and two-photon photoelectron spectroscopy (2PPE) for the HB-HBC thin film. In combination with an approximate value of the polarization energy of 1.7 eV obtained for many polycyclic aromatic hydrocarbons (PAHs)[20], the E_i and EA of the HB-HBC molecules were evaluated. Supplementary Fig. 10 shows the UPS spectrum for an HB-HBC thin film, wherein the HOMO level was observed at 5.9 eV from the vacuum level. On the other hand, Supplementary Fig. 11 shows the 2PPE spectra for the HB-HBC thin film, wherein the LUMO level was observed at 2.9 eV from the vacuum level. Since the polarization energy, i.e., the stabilization energy of ions by the surrounding molecules in the thin film, is 1.7 eV for typical PAHs, the E_i and EA values for an HB-HBC molecule were determined to be 7.6 eV and 1.2 eV, respectively. These values are consistent with the experimental and calculated E_i values as well as the calculated EA values for the HBC molecules obtained using DFT calculations (B3LYP/6-311+G(d,p) level), as listed in Supplementary Table 2.

These values give endothermic dissociation limits for the corresponding cations and anions, ΔE (Supplementary Table 3); the values used for this estimation are underlined in Supplementary Table 2. On

C_{60}, therefore, it is favorable for both Al_{13} and B@Al_{12} to become cations combined with C_{60} anions, because their ΔE values are smaller than those for the opposite charge combinations. On HB-HBC, however, the anions of Al_{13} and B@Al_{12} more favorably combine with the HB-HBC cations due to the smaller ΔE values resulting from these interactions. The HB-HBC molecule exhibits an opposite behavior to that of C_{60} because the E_i value of HB-HBC is smaller than that of C_{60}, and because the EA value of HB-HBC is 1 eV lower than that of C_{60}.

By comparing the absolute values of ΔE, it can be seen that the ΔE for B@Al_{12} on HB-HBC is larger (4.47 eV) than the other values, because CT complexation overcomes the endothermic energy gap even between B@Al_{12} on HB-HBC. In other words, the stabilization due to CT between B@Al_{12} and HB-HBC is considered larger than 4.47 eV. Considering a point charge model, when the positive charge and the negative charge approach to a distance (r) of 3 Å, the stabilization energy ($= e^2/((4\pi\varepsilon_0)r)$) is calculated to be 4.8 eV, which is actually larger than the endothermic gap between B@Al_{12} and HB-HBC. Moreover, the CT complex on a C_{60} or HB-HBC substrate is further stabilized by the polarization of the C_{60} or HB-HBC surroundings. On the C_{60}/HB-HBC substrate, the polarization energy of charged C_{60}/HB-HBC on the topmost is estimated to be ~0.85 eV, which is half of the polarization energy of the corresponding negative or positive polaron in the organic film (1.7 eV). Furthermore, similar to well-known CT complexes[21,22], the presence of covalent bonds due to molecular orbital overlapping should also contribute to the interactions between Al_{13}/B@Al_{12} and C_{60}/HB-HBC, thereby allowing the endothermic gap to be seemingly overcome.

This study shows that by covering the surface with organic molecules, the molecularly localized interactions with the nanoclusters are emphasized, and the cluster-surface interactions can be designed by tuning of the physical properties of the deposited organic molecule. Although it is essential to select a suitable substrate, designing the interaction by the appropriate selection of an organic molecule is a more strategic means to fabricate nanoscale heterointerfaces and nanocluster-based assemblies on a surface.

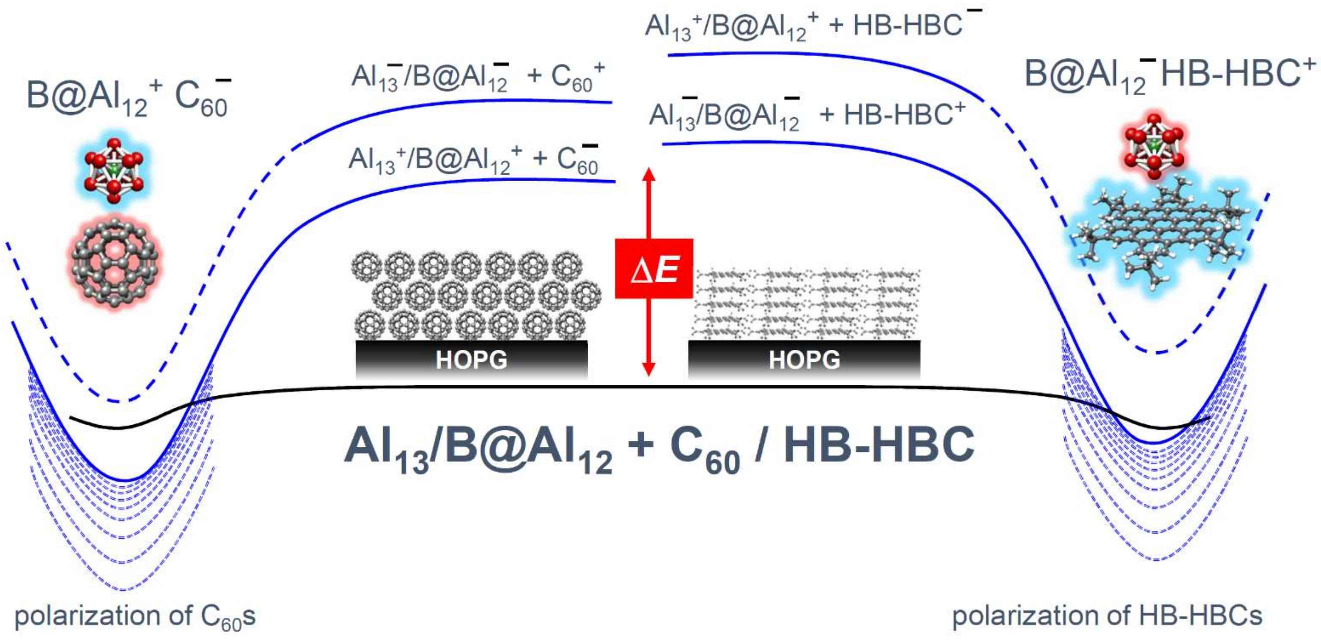

Supplementary Fig. 12 Schematic potential curves for the complexation between Al_{13}/B@Al_{12} and C_{60}/HB-HBC. When B@Al_{12} is deposited onto C_{60} (left-hand side) or HB-HBC (right-hand side) molecular layers, charge transfer molecularly takes place between them, where the charged C_{60} or HB-HBC is further stabilized by the polarization of the surroundings. The energy gap, ΔE, represents the endothermic dissociation limits of the corresponding cations and anions against the neutral B@Al_{12} and C_{60} or HB-HBC. Instead of B@Al_{12}, these schematic energetic representations can also be applied to Al_{13}.

Supplementary Table 2 Experimental and calculated ionization energies (E_i) and electron affinities (EA) of Al_{13}, $B@Al_{12}$, C_{60}, and HB-HBC in eV. The underlined values are used for estimation purposes.

Superatom	E_i (calc)	E_i (exp)	EA (calc)	EA (exp)
Al_{13}	<u>6.42</u> ref.14	~ 6.42 ref.23	3.57 ref.16	<u>3.62</u> ref.11
$B@Al_{12}$	<u>6.64</u> ref.15	6.42< E_i <7.90 ref.13	3.19 ref.17	<u>3.13</u> ref.12

Molecule	E_i (calc)	E_i (exp)	EA (calc)	EA (exp)
C_{60}	----	<u>7.57</u> ref.18	----	<u>2.683</u> ref.19
HBC	6.57 [a)]	6.87 ref.24	1.21 [a)]	----
HB-HBC	----	<u>7.6</u> [b)]	----	<u>1.2</u> [b)]

a) DFT calculations (B3LYP/6-311+G(d,p) level).

b) The experimental E_i and EA values for HB-HBC are evaluated from HOMO +1.7 eV and LUMO −1.7 eV, respectively (see text in Supplementary Note 5).

Supplementary Table 3 Calculated endothermic dissociation limits of the corresponding cations and anions (Δ*E*) against neutral Al_{13}/B@Al_{12} and organic molecules in eV. The smaller values indicated in bold represent favorable CT channel.

Superatom (SA)	$SA^+ C_{60}^-$		$SA^- C_{60}^+$
Al_{13}	**3.74**	<	3.95
B@Al_{12}	**3.96**	<	4.44

Superatom (SA)	SA^+ HB-HBC$^-$		SA^- HB-HBC$^+$
Al_{13}	5.22	>	**3.98**
B@Al_{12}	5.44	>	**4.47**

Supplementary Table 4 Cartesian coordinates for Al_{13}^{-}, B@Al_{12}^{-}, Al_{13}^{+}, and B@Al_{12}^{+}

Coordinate of Al_{13}^{-} (Symmetry I_h).

Al1	1.837128	1.214993	-1.486674
Al2	1.946997	-1.527916	-0.967703
Al3	-0.065017	-0.496224	-2.609144
Al4	-0.851575	1.972175	-1.563638
Al5	-2.403337	-0.302551	-1.092401
Al6	-0.674110	-2.465771	-0.724584
Al7	-1.837445	-1.214794	1.486036
Al8	0.851033	-1.972809	1.563572
Al9	2.403663	0.302553	1.092796
Al10	0.064696	0.495927	2.609596
Al11	0.674207	2.466241	0.724210
Al12	-1.946691	1.528059	0.967632
Al13	0.000452	0.000117	0.000301

Coordinate of B@Al_{12}^{-} (Symmetry I_h)

Al1	-1.393138	0.799781	1.973172
Al2	-2.094013	-1.346454	0.535553
Al3	0.074845	-1.432305	2.098974
Al4	1.277595	0.953581	1.982982
Al5	2.230856	-1.097146	0.551407
Al6	0.146338	-2.515922	-0.344421
Al7	1.393555	-0.799125	-1.972716
Al8	-1.278077	-0.953000	-1.982600
Al9	-2.227818	1.095099	-0.549686
Al10	-0.074934	1.434070	-2.102415
Al11	-0.146422	2.517579	0.343816
Al12	2.091228	1.344073	-0.533909
B1	-0.000040	-0.000601	-0.000404

Coordinate of Al_{13}^{+} (Symmetry C_1)

Al1	-2.562505	0.475359	-1.287535
Al2	2.044835	-1.158681	1.107529
Al3	-2.272039	-0.587318	1.110940
Al4	-1.169118	1.909599	1.341803
Al5	-0.018918	-0.161348	2.349839
Al6	-0.976973	2.520900	-1.181339
Al7	1.024482	-2.275151	-1.419803
Al8	2.599446	-0.192192	-1.285018

Al9	-1.577369	-1.942676	-1.415137
Al10	-0.312049	-2.377028	0.968113
Al11	1.629379	1.536988	1.332790
Al12	1.583769	2.187785	-1.189979
Al13	0.007059	0.063764	-0.432201

Coordinate of B@Al_{12}^+ (Symmetry C_i)

Al1	-0.471268	1.638699	1.693579
Al2	-2.190736	-0.336752	1.404181
Al3	0.385250	-0.699134	2.235031
Al4	2.137921	0.944724	1.182092
Al5	2.022561	-1.597269	0.411029
Al6	-0.660245	-2.294208	0.523005
Al7	0.471210	-1.638646	-1.693521
Al8	-2.137850	-0.944678	-1.182047
Al9	-2.022528	1.597230	-0.411044
Al10	-0.385242	0.699186	-2.235161
Al11	0.660225	2.294106	-0.522964
Al12	2.190716	0.336739	-1.404177
B1	-0.000035	0.000003	-0.000010

Supplementary References

1. Rathore, R. & Burns, C. L. A practical one-pot synthesis of soluble hexa-*peri*-hexabenzocoronene and isolation of its cation-radical salt. *J. Org. Chem.* **68**, 4071–4074 (2003).
2. Nakaya, M., Iwasa, T., Tsunoyama, H., Eguchi, T. & Nakajima, A. Formation of a superatom monolayer using gas-phase-synthesized $Ta@Si_{16}$ nanocluster ions. *Nanoscale* **6**, 14702–14707 (2014).
3. Shibuta, M., Ohta, T., Nakaya, M., Tsunoyama, H., Eguchi, T. & Nakajima, A. Chemical characterization of an alkali-like superatom consisting of a Ta-encapsulating Si_{16} cage. *J. Am. Chem. Soc.* **137**, 14015–14018 (2015).
4. Ohta, T., Shibuta, M., Tsunoyama, H., Eguchi, T. & Nakajima, A. Charge transfer complexation of Ta-encapsulating $Ta@Si_{16}$ superatom with C_{60}. *J. Phys. Chem. C* **120**, 15265–15271 (2016).
5. Koyasu, K., Atobe, J., Akutsu, M., Mitsui, M. & Nakajima, A. Electronic and geometric stabilities of clusters with transition metal encapsulated by silicon. *J. Phys. Chem. A* **111**, 42–49 (2007).
6. Koyasu, K., Atobe, J., Furuse, S. & Nakajima, A. Anion photoelectron spectroscopy of transition metal- and lanthanide metal-silicon clusters; MSi_n^- (n = 6-20). *J. Chem. Phys.* **129**, 214301 (2008).
7. Doniach, S. & Šunjić, M. Many-electron singularity in X-ray photoemission and X-ray line spectra from metals. *J. Phys. C: Solid St. Phys.* **3**, 285–291 (1970).
8. Ohta, T., Shibuta, M., Tsunoyama, H., Eguchi, T. & Nakajima, A. Charge transfer complexation of Ta-encapsulating $Ta@Si_{16}$ superatom with C_{60}. *J. Phys. Chem. C* **120**, 15265–15271 (2016).
9. Bianconi, A., Bachrach, R. Z., Hagstrom, S. B. M. & Flodström, Al-$A1_2O_3$ interface study using surface soft-x-ray absorption and photoemission spectroscopy. *Phys. Rev. B* **19**, 2837–2843 (1973).
10. Levine, R. D. & Bernstein R. D. Molecular reaction dynamics and chemical reactivity. *Oxford Univ. Press,* **1987**.
11. Li, X., Wu, H., Wang, X.-B. & Wang, L.-S. s-p Hybridization and electron shell structures in aluminum clusters: a photoelectron spectroscopy study. *Phys. Rev. Lett.* **81**, 1909–1912 (1998).
12. Kawamata, H., Negishi, Y., Nakajima, A. & Kaya, K. Electronic properties of substituted aluminum clusters by boron and carbon atoms ($Al_nB_m^-/Al_nC_m^-$); New insights into s-p hybridization and perturbed shell structures. *Chem. Phys. Lett.* **337**, 255–262 (2001).
13. Akutsu, M., Koyasu, K., Atobe, J., Hosoya, N., Miyajima, K., Mitsui, M. & Nakajima, A. Experimental and theoretical characterization of aluminum-based binary superatoms of $Al_{12}X$ and their cluster salts. *J. Phys. Chem. A* **110**, 12073–12076 (2006).
14. Akola, J., H. Häkkinen, H. & Manninen, M. Ionization potential of aluminum clusters. *Phys. Rev. B* **58**, 3601–3604 (1998).
15. Chauhan, V., Reber, A. C. & Khanna, S. N. Strong lowering of ionization energy of metallic clusters by organic ligands without changing shell filling. *Nat. Commun.* **9**, 2357 (2018).
16. Electron affinity of Al_{13}: a correlated electronic structure study. Smith, Q. A. & Gordon, M. S. *J. Phys. Chem. A* **115**, 899–903 (2011).
17. Wan, J. & Fournier, R. Why is $Al_{11}B_2^-$ not a magic number in TOF-MS? *J. Chem. Phys.* **119**, 5949–5954 (2003).
18. Yoo, R. K., Ruscic, B. & Berkowitz, J. Vacuum ultraviolet photoionization mass spectrometric study of C_{60}. *J. Chem. Phys.* **96**, 911–918 (1992).
19. Huang, D. L., Dau, P. D., Liu, H. T. & Wang, L. S. High-resolution photoelectron imaging of cold C_{60}^- anions and accurate determination of the electron affinity of C_{60}. *J. Chem. Phys.* **140**, 224315 (2014).
20. Sato, N., Seki, K. & Inokuchi, H. Polarization energies of organic solids determined by ultraviolet photoelectron spectroscopy. *J. Chem Soc., Faraday Trans. 2* **77**, 1621–1633 (1981).
21. Mulliken, R. S. Molecular compounds and their spectra. II. *J. Am. Chem. Soc.* **74**, 811–824 (1952).
22. Mulliken, R. S. Molecular compounds and their spectra. III. The interaction of electron donors and acceptors. *J. Phys. Chem.* **56**, 801–822 (1952).
23. Schriver, K. E., Persson, J. L., Honea, E. C. & Whetten, R. L. Electronic shell structure of group-IIIA metal atomic clusters. *Phys. Rev. Lett.* **64**, 2539, (1990).
24. Clar, E. & Schmidt, W. Correlations between photoelectron and ultraviolet absorption spectra of polycyclic hydrocarbons. The terrylene and peropyrene series. *Tetrahedron,* **33**, 2093–2097 (1997).
